# A Survey on AI Techniques for Thoracic Diseases Diagnosis Using Medical Images

**DOI:** 10.3390/diagnostics12123034

**Published:** 2022-12-03

**Authors:** Fatma A. Mostafa, Lamiaa A. Elrefaei, Mostafa M. Fouda, Aya Hossam

**Affiliations:** 1 Department of Electrical Engineering, Faculty of Engineering at Shoubra, Benha University, Cairo 11672, Egypt; 2 Department of Electrical and Computer Engineering, College of Science and Engineering, Idaho State University, Pocatello, ID 83209, USA

**Keywords:** thoracic diseases, deep learning, transfer learning, ensemble learning, CXR

## Abstract

Thoracic diseases refer to disorders that affect the lungs, heart, and other parts of the rib cage, such as pneumonia, novel coronavirus disease (COVID-19), tuberculosis, cardiomegaly, and fracture. Millions of people die every year from thoracic diseases. Therefore, early detection of these diseases is essential and can save many lives. Earlier, only highly experienced radiologists examined thoracic diseases, but recent developments in image processing and deep learning techniques are opening the door for the automated detection of these diseases. In this paper, we present a comprehensive review including: types of thoracic diseases; examination types of thoracic images; image pre-processing; models of deep learning applied to the detection of thoracic diseases (e.g., pneumonia, COVID-19, edema, fibrosis, tuberculosis, chronic obstructive pulmonary disease (COPD), and lung cancer); transfer learning background knowledge; ensemble learning; and future initiatives for improving the efficacy of deep learning models in applications that detect thoracic diseases. Through this survey paper, researchers may be able to gain an overall and systematic knowledge of deep learning applications in medical thoracic images. The review investigates a performance comparison of various models and a comparison of various datasets.

## 1. Introduction

Thoracic diseases are diseases of the organs within the rib cage, including heart and lung diseases. Lung diseases result in hypoxia and dyspnea. Furthermore, some diseases may cause the failure of the entire respiratory system and thus lead to death [1], such as the novel coronavirus disease (COVID-19), which emerged recently and became a pandemic that poses a threat to the entire world. There are several types of thoracic diseases [2,3] represented as follows: (*i*) asthma, COVID-19, tuberculosis (TB), and chronic obstructive pulmonary disease (COPD) are examples of diseases that affect the airways or lungs; (ii) diseases that affect the heart, such as cardiomegaly and heart failure; (iii) other diseases affecting bones and muscles in the chest, such as fracture and lung metastasis. The World Health Organization (WHO) has classified pneumonia as the third-deadliest disease in the world after heart disease and cerebral palsy. As in 2019, 2.5 million death cases from pneumonia around the world [4], 14% of all deaths of children under five years old, which results in the death of 672,000 children [5]. In addition, COVID-19 has caused more than 6.5 million death cases around the world since its emergence in 2019 [6]. Tuberculosis (TB) resulted in the death of approximately 1.5 million people in 2020.

*Early detection* refers to detecting symptomatic patients as early as possible, *detection* refers to the act of detecting or sensing something; discovering something that was hidden or disguised, and *diagnosis* refers to the identification of the nature and cause of an illness. As a result, human health is at a serious risk due to thoracic diseases, and early detection of these diseases improves recovery and reduces mortality. The consultant provided by a thoracic expert or radiologist is solely responsible for the patient’s diagnosis. However, there may be emergency situations where radiology professionals are too busy, unavailable, or unable to diagnose a large number of thoracic images rapidly [7,8]. Artificial intelligence (AI) systems can be extremely useful in this regard [9].

AI is used to analyze, display, and understand complex medical and health data. The ability of computer algorithms to guess conclusions based solely on input data are known as artificial intelligence. The major goal of health-related AI applications is to figure out how clinical procedures affect patient outcomes. AI systems are employed in diagnostics, treatment protocol creation, drug research, personalized medicine, patient monitoring, and care. What distinguishes AI technology from traditional healthcare solutions is its ability to collect and process data and deliver a specific and fast result [10]. Artificial intelligence does this through the use of machine learning (ML) and deep learning (DL) algorithms. These processes are able to recognize patterns of behavior and develop their own logic.

ML allows software applications to become more accurate at predicting outcomes without being explicitly programmed to do so. Machine learning algorithms use historical data as input to predict new output values. There are four basic approaches for ML: supervised learning, unsupervised learning, semi-supervised learning, and reinforcement learning [11]. The type of the algorithm; that the data scientists choose to use, depends on what type of data they want to predict. There are different learning techniques which can be summarized as follows:

- **Supervised learning**: data scientists supply algorithms with labeled training data and define the variables they want the algorithm to assess for correlations. Both the input and the output of the algorithm are specified. Some of the most common algorithms in supervised learning include Support Vector Machines (SVM), Decision Trees, and Random Forest;
- **Unsupervised learning**: involves algorithms that train on unlabeled data. The algorithm scans through datasets looking for any meaningful connection. The data that the algorithms train on as well as the predictions or recommendations they output are predetermined;
- **Semi-supervised learning**: occurs when part of the given input data has been labeled. Unsupervised and semi-supervised learning can be more appealing alternatives as it can be time-consuming and costly to rely on domain expertise to label data appropriately for supervised learning;
- **Reinforcement learning**: data scientists typically use reinforcement learning to teach a machine to complete a multi-step process for which there are clearly defined rules. Data scientists program an algorithm to complete a task and give it positive or negative cues as it works out how to complete a task. However, for the most part, the algorithm decides on its own what steps to take along the way.

Table 1 illustrates a simplified summary of the four types of ML approaches.

Machine learning models are trained using large amounts of input data in order to provide relevant insights and predictions. Currently, several datasets of thoracic images for different thoracic diseases are publicly available. The doctor’s efficiency can be improved by AI systems, especially through DL.

AI is now widely applied in a variety of sectors, including medicine and the rapid detection of diseases. AI played a key contribution in developing a Coronavirus vaccine in record time [12]. In South Korea, intelligence assisted doctors in learning the statistics of affected persons, allowing them to predict the Coronavirus outbreak at the start of the crisis. At a time when many governments around the world were still considering the idea of imposing a blanket closure owing to the pandemic, a business in Seoul (Seegene) employed artificial intelligence to develop tests to detect the Coronavirus in weeks, whereas it would have taken months without it [13]. “People thought the delta mutant would spread across the African continent, clogging hospitals with patients, but with AI, we can control it,” explains a South African AI expert. Artificial intelligence has aided scientists in gaining a better understanding of how quickly the virus can change, as well as in developing and testing vaccines against the Coronavirus. As indicated in the paper, many research has demonstrated the value of AI algorithms in detecting specific types of diseases with great accuracy. The initial use of AI in the face of a health catastrophe is almost definitely to assist researchers in developing a vaccine that will protect caretakers while also containing the pandemic. Biomedicine and research rely on a wide range of methodologies, many of which have long been aided by computer science and statistics applications. As a result, AI is a component of this continuity [14]. Scientists have already avoided months of experimentation by using AI to forecast the virus structure. Even if it is constrained due to so-called “continuous” rules and infinite combinatorics for the study of protein folding, AI appears to have provided important support in this regard. Moderna, an American start-up, has made a name for itself by mastering biotechnology based on messenger ribonucleic acid (mRNA), which necessitates the study of protein folding. With the use of bioinformatics, of which AI is a key part, it was able to dramatically cut the time it took to build a prototype vaccine that could be tested on humans. In February 2020, Baidu, a Chinese technological powerhouse, released its Linearfold prediction algorithm in collaboration with Oregon State University and the University of Rochester to research the same protein folding. This method predicts the structure of a virus’ secondary ribonucleic acid (RNA) significantly faster than standard algorithms, giving scientists more knowledge on how viruses spread. Linearfold could have predicted the secondary structure of COVID-19’s RNA sequence in 27 s instead of 55 min. DeepMind, a subsidiary of Alphabet, the parent company of Google, has also shared its coronavirus protein structure predictions with its AlphaFold AI system. IBM, Amazon, Google, and Microsoft have also offered their servers’ computing power to the US government for the processing of very big datasets in epidemiology, bioinformatics, and molecular modeling.

Thus, an intelligent and automatic system is required to diagnose the hidden patterns in clinical data. Using image classification and detection techniques, DL models have provided advanced digital imaging applications for faster disease detection [15]. DL is based on extracting features from raw data using multiple layers to recognize different parts of the input data [16]. DL provides guidance to doctors and other researchers on how to automatically detect diseases [17]. However, DL requires a large amount of data to perform better. Therefore, images will be crucial data for detecting diseases [18].

Deep learning use cases in healthcare such as medical imaging, data analytic, personalized medical treatments, drug discovery, genomics analysis, and mental health research. Among the recent work based on deep learning are the detection of acute lymphoblastic leukemia [19,20,21,22,23,24], chest disease [25], and Alzheimer’s disease [26,27,28].

Although AI has made an effective contribution to the medical field, it has some limitations [29] as:

1. Cost (analyzing data will be very costly both in terms of energy and hardware use);
2. These technologies are still a group of very rapidly developing technologies, and therefore they are still under development. Thus, we need experts in this field to deal with it;
3. These technologies are not widely available in the healthcare sector;
4. Security needs to be integral in the AI process.

The primary contributions to this article are as follows:

1. It provides a comprehensive overview of the use of AI in detecting thoracic diseases, including COVID-19;
2. It presented the different types of AI models that used to detect thoracic diseases and the databases that include those diseases. In addition, the progress of the works and the direction the researchers are moving in this domain throughout the recent years;
3. To express that CNN has penetrated the field of understanding the medical picture with high accuracy;
4. It collected many different databases for thoracic diseases with descriptions;
5. It also presents the issues of thoracic diseases detection using deep learning found in the literature studies.

## 2. Methodology

The methodology used to conduct the survey of recent thoracic diseases detection using DL/ML models: First, an appropriate dataset containing a large number of images that includes related diseases must be selected, and this is described in detail in the next section. Second, the DL/ML algorithms that are applied to detect related diseases must be identified, and this is described in detail in the next section. In the last stage, the performance of the model used in the detection of the disease is determined.

## 3. The Taxonomy of State-of-the-Art Work on Thoracic Diseases Detection Using DL/ML

In this section, a taxonomy of the recent work on thoracic diseases detection using DL/ML is presented, which is the first contribution of this paper.

The taxonomy consists of 9 traits that are common in the surveyed articles: image type, dataset description, image pre-processing, deep learning models, ways to train deep learning, the ensemble of classifiers, pre-trained models, type of disease, and evaluation criteria.

### 3.1. Imaging Thoracic Exams

Medical and health protocols recommend thoracic imaging because it is a rapid and painless technique. Infected patients, including children, adults, and the elderly, are now being assessed using image scans. Imaging systems that rely on AI technologies are provided with thousands of medical images so that these systems can identify abnormal masses that could indicate the onset of disease. Then, these systems are able to identify a specific area on the radiograph for the doctor to examine with greater accuracy, thus integrating ‘artificial intelligence’ techniques with the doctors’ efforts [30]. Some diseases of the thoracic require radiological images (as X-ray or CT-Scan) to detect the disease. Others require examination of the tissues themselves to confirm the presence of the disease. The most examination types to diagnose thoracic diseases [31] are:

- Chest X-ray (CXR): can be used to check for diseases such as pneumonia [32] and a lung infection that causes fluid buildup [33]. It can also be used to detect cancer or pulmonary fibrosis, which is a scar tissue buildup in the lungs. CXR scans are commonly used in clinical practice since they are inexpensive, simple to perform, give a quick scan for the patient as two-dimensional (2D) images, and can be widely used for diagnosis and treatment of lung and cardiovascular diseases [34,35]. Although X-rays are frequently used, they have side effects such as exposure to ionizing radiation harmful to the human body and relatively low information when compared to other imaging methods;
- Computerized Tomography (CT): is a more advanced imaging test that can be used to detect disorders such as cancer that an X-ray could miss [36,37,38,39]. A CT scan is a series of X-rays taken from various angles that are patched together to create a complete image. While CT scans are more reliable in diagnosing COVID-19, they are less accurate in diagnosing non-viral pneumonia-like consolidation [40]. The CT scan is very accurate spatial information and quick, but the disadvantages of the CT scan are the risk of exposure to radiation is high, require expensive equipment, and is therefore not always accessible to all levels of people;
- Histopathology: often known as histology, is the microscopic examination of organic tissues in order to observe the appearance of diseased cells [41]. The tissue that was sent for testing, as well as the characteristics of the tumor under the microscope is described in a histopathology report [42]. A biopsy report or a pathology report are both terms used to describe a histopathology report. It can identify features of what cancer looks like under the microscope, or detect cardiomegaly disease [43]. Histology examination is low cost and allows an evaluation of infection distribution in various tissues. However, it needs 2–7 days of preparation time, might not detect low-level infection, and it depends on the expertise of pathologists;
- Sputum Smear Microscopy: refers to the microscopic investigation of sputum [44]. This has been proved to be one of the most effective ways of detecting tuberculosis infection in patients so that treatment can begin [45]. In some times, a chest X-ray and a sputum sample are needed to find out if a person has tuberculosis [46]. In poor and middle-income countries, sputum smear microscopy has been the major approach for diagnosing pulmonary tuberculosis [47]. Sputum smear microscopy examination has a long experience, inexpensive, and is used for the follow-up of patients on treatment. However, it is cumbersome for laboratory staff and patients and needs two samples;
- Magnetic Resonance Imaging (MRI): is a type of scan that uses powerful magnetic fields and radio waves to provide detailed images of the inside of the body. An MRI scanner is a huge tube with powerful magnets within. During the scan, the patient will be lying inside the tube. MRI scans can be used to investigate practically any region of the body, including the brain, breast, and heart problems [48]. MRI has more advantages as a 3D technique and is safer (no ionizing radiation, and excellent soft-tissue contrast. However, it has long total scan times (30–75 min), is not as readily accessible, and is claustrophobic (enclosed space).

### 3.2. Dataset Description

A high number of trainable parameters are required to train a neural network model, which necessitates very large datasets. Several publicly available open-source datasets of thoracic images are reported in Table 2. Some of the datasets contain images of multiple diseases of the thoracic, such as the National Institute of Health (NIH) dataset [49], ChestX-ray8 [49], Chowdhury’s Kaggle [50], COVID-19 Image Data Collection [51], PadChest [52], CheXpert [53], COVIDx Dataset [54], COVID-19 Radiography Database [55], and the MIMIC dataset [56].

Other datasets contain only images of one disease, such as Andrew’s Kaggle dataset, JSRT [57], Optical Coherence Tomography (OCT) and Chest X-ray Images [58], RSNA Pneumonia Detection Challenge Dataset [59], RIH-CXR [60], NCI Genomic Data Commons [61], COVID Chest X-ray Dataset, ImageCLEF [62], ChestX-ray images (Pneumonia) [58], Montgomery and Shenzhen datasets [63], Shenzhen datasets [63], COVID-CT Dataset [64], Autofocus database [65], Sajid’s Kaggle dataset, LDOCTCXR [66], Sunnybrook Cardiac MRI dataset [67], CPTAC-LUAD dataset [68], and the LIDC-IDRI dataset [69].

All of these datasets display about 32 different diseases labels which can listed as follows: Pneumonia, Viral Pneumonia, Bacterial Pneumonia, Atypical Pneumonia, COVID-19, Edema, Consolidation, Atelectasis, Lesion, Asbestosis Signs, Cardiomegaly, Enlarged Cardiomediastinum, Heart Insufficiency, Pleural Thickening, Pneumothorax, Fracture, Lung Metastasis, Mass, Hernia, Effusion, Nodule, Emphysema, Fibrosis, COPD signs, Tuberculosis, Tuberculosis Sequelae, Post Radiotherapy Changes, Pulmonary Hypertension, Respiratory Distress, Lymphangitis Carcinomatosa, Infiltration, and Lepidic Adenocarcinoma. The majority of the available datasets are CXR images, as shown in Table 2.

**Table 2:** Public datasets are used by contributions to deep learning applications in pulmonary medical imaging analysis.

Name of Dataset/Ref. & Download Link	Dataset Classes	Images Type	Dataset Description
ChestX-ray8 [49,70]	8 thoracic diseases and a normal case. Diseases labels are Atelectasis, Cardiomegaly, Effusion, Infiltration, Mass, Nodule, Pneumonia, and Pneumothorax.	X-ray	108,948 frontal images in PNG format with resolution of images 1024 × 1024, from 30,805 patients.
ChestX-ray14 [49,70]	14 thoracic diseases and a normal case. Diseases labels are Edema, Cardiomegaly, Effusion, Infiltration, Mass, Nodule, Pneumonia, Pneumothorax, Atelectasis, Hernia, Pleural thickening, Emphysema, Fibrosis, and Consolidation.	X-ray	112,120 total images in PNG format from 32,717 patients. Images resolution 1024 × 1024.
ImageCLEF 2019 [39,71]	Tuberculosis	CT	335 images in PNG format for 218 patients, with a set of clinically relevant metadata. Image size 512 × 512 pixels.
ImageCLEF 2020 [62,72]	Tuberculosis	CT	403 images in PNG format 512 × 512 pixels.
JSRT dataset [57,73]	Normal and Lung Nodules	CT and X-ray	93 normal and 154 nodule images in PNG format, with metadata. Image size 2048 × 2048 pixels.
Montgomery dataset [63,74]	Tuberculosis and Normal	X-ray	138 TB images and 80 normal images in PNG format with metadata. Images size is either 4020 × 4892 or 4892 × 4020 pixels.
Autofocus database [65,75]	Tuberculosis	Sputum Smear Microscopy	1200 images with resolution of 2816 × 2112 pixels.
Andrew’s Kaggle Database [76]	COVID-19	CT and X-ray	16 CT images and 79 X-ray images in JPEG format with different size of images.
Chowdhury’s Kaggle dataset [50,77]	COVID-19, Pneumonia, and Normal	X-ray	1341 Normal, 219 COVID-19, and 1345 Pneumonia in PNG format images.
Optical Coherence Tomography (OCT) and Chest X-ray Images [58,78]	Normal and Pneumonia	X-ray and CT	5856 images, 1583 normal and 4273 pneumonia images in JPEG format with different images size. Bacterial Pneumonia, Viral Pneumonia, and COVID-19 are all represented in the Pneumonia class.
Shenzhen dataset [63,79]	Tuberculosis and normal	X-ray	662 frontal images; 326 Normal and 336 TB. Images are in PNG format with different size about 3000 × 3000.
CheXpert [53,80]	18 different diseases labels as Atelectasis, Consolidation, Infiltration, Pneumothorax, Edema, Emphysema, Fibrosis, Effusion, Pneumonia, Pleural Thickening, Cardiomegaly, Nodule, Mass, Hernia, Lung Lesion, Fracture, Lung Opacity, and Enlarged Cardiomediastinum	X-ray	224,316 images in PNG and JPG format from 65,240 patients with both frontal and lateral views, with different images size.
RSNA Dataset [59]	Pneumonia and Normal	X-ray	5863 images in JPEG format with different images size.
PadChest [52,81]	16 different diseases labels as Pulmonary Fibrosis, COPD signs, Pulmonary Hypertension, Pneumonia, Heart Insufficiency, Pulmonary Edema, Emphysema, Tuberculosis, Tuberculosis Sequelae, Lung Metastasis, Post Radiotherapy Changes, Atypical Pneumonia, Respiratory Distress, Asbestosis Signs, Lymphangitis Carcinomatosa, and Lepidic Adenocarcinoma	X-ray	160,868 images in PNG format with different images size from 67,625 patients and 206,222 reports.
NCI Genomic Data Commons [61,82]	Lung Cancer	Histopathology	More than 575,000 images with size 512 × 512.
Covid Chest X-ray database [83]	COVID-19	X-ray	231 COVID-19 images in JPEG format with different images size, and contains metadata
RIH-CXR [60]	Normal and Abnormal	X-ray	17,202 frontal images; 9030 Normal and 8172 abnormal images from 14,471 patients. It also contains metadata.
Sajid’s Kaggle database [84]	Normal and COVID-19	X-ray	28 Normal and 70 COVID-19 images in JPEG, JPG, and PNG format with different images size.
Covid-19 Radiography Database [55,77]	Normal, COVID-19, Lung Opacity, and Viral Pneumonia	X-ray	10200 Normal, 3616 COVID-19, 6012 Lung Opacity, and 1345 Viral Pneumonia. 299 × 299 pixels images in PNG format. The dataset contains metadata.
ChestX-ray images (Pneumonia) [58,85]	Normal and Pneumonia	X-ray	5232 chest X-ray images from children. 3883 pneumonia (2538 bacterial and 1345 Viral) and 1349 normal, from a total of 5856 patients to train a model and then tested with 234 normal and 390 Pneumonia from 624 patients. The images are in JPEG format with different size.
COVID-CT database [64,86]	Normal and COVID-19	CT	15589 images for normal and 48260 images for COVID-19 in DICOM format with 512×512 pixels.
COVID-19 Image Data Collection [51,87]	4 classes: COVID-19, Viral Pneumonia, Bacterial Pneumonia, and Normal	X-ray	It contains 306 images, 79 images for normal, 69 images for COVID-19, 79 images for Bacterial Pneumonia, and 79 images for Viral Pneumonia in JPG format with different size. It also contains metadata.
LIDC-IDRI [69,88]	Lung Cancer	CT	It conatins 1018 images from 1010 patients. It also contains metadata.
LDOCTCXR [66,78]	Normal and Pneumonia	X-ray	3883 Pneumonia and 1349 Normal images.
COVIDx Dataset [54,89]	Pneumonia, Normal, and COVID-19	X-ray	5559 Pneumonia, 8066 Normal, and 573 COVID-19 images
CPTAC-LUAD Dataset [68,90]	Lung Cancer	MRI, CT, and X-ray	43,420 images in DICOM format.
Sunnybrook Cardiac MRI [67,91]	Heart Disease	MRI	The SCD had 45 MRI images with the combination of patients with the following classes such as healthy, hypertrophy, heart failure with infarction and heart failure without infarction. The image resolution is 255 × 255.
MIMIC-CXR Dataset [56,92]	Chest radiograph	X-ray	377,110 chest radiographs with 227,835 radiology reports in DICOM format. The size of the chest radiographs varies and is around 3000 × 3000 pixels.

### 3.3. Image Pre-Processing

**Figure 1:**
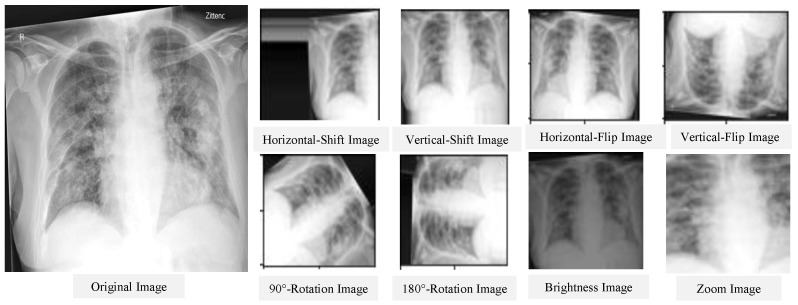
Results of the data augmentation procedure on a certain CXR image.

The main goal of image pre-processing a segmentation is to enhance the quality of the input image and reduce the amount of noise. Images must match the network’s input size in order to train a network model and make predictions on new data. You can re-scale or crop your data to the desired size if you need to modify the size of your images to fit the network. By using random augmentation to your data, you can effectively enhance the amount of training data [93]. The image augmentation creates many variations from the original images. The image augmentation process may include cropping, flipping, brightness, saturation, contrast, rotation, scaling, translation, zooms, and/or adding noise, as shown in Figure 1. The figure illustrates the different variations from the input X-ray image, including horizontal and vertical shift, horizontal and vertical flip, rotation, brightness, and zoom using Keras ImageDataGenerator method. As an example of data augmentation pre-processing, in [94], the authors used data augmentation to diagnose pneumonia disease and achieved an accuracy of 96.61%. For image classification tasks, in terms of training loss, accuracy, and validation loss, a deep learning model with image augmentation outperforms a deep learning model without it. Augmentation can solve the problem of imbalanced classes in binary classification [95]. When training a binary classification model, the resulting model will be biased if one class has more samples than the other. There are other advanced methods that are used to handle the imbalanced dataset, such as the synthetic minority oversampling technique (SMOTE) [96], generative adversarial networks (GAN) [97,98], and adaptive synthetic sampling method (ADAYSN) [99]. In [98], the authors used GAN method to detect lung cancer and achieved an accuracy of 99.86%. Image segmentation is used to perform operations on images to detect patterns and retrieve information from them. Image segmentation is the process of splitting a digital image into several regions or objects, each of which is made up of sets of pixels with similar features or attributes that are labeled differently to represent distinct regions or objects. The purpose of segmentation is to make an image more understandable and easier to evaluate by simplifying and/or changing its representation, as in [100], which achieved an AUC of 97.7% for segmentation. In [101], the authors achieved an accuracy of 96.47% without segmentation and 98.6% with segmentation.

### 3.4. Deep Learning Models

Deep learning has become very popular in the field of scientific computing because its algorithms are widely used to solve complex problems in medical applications. Deep learning algorithms employ several types of neural networks to perform specific tasks such as speech recognition, image recognition, data compression, machine translation, data visualization, and image classification. Deep learning supports the classification, quantification, and identification of medical images. DL is a learning type of neural network relevant to layer size [102], and it refers to systems that learn from experience on large data sets. Deep learning is predicated on the concept of extracting features from input data utilizing many layers to find different elements that are important to the input data. Data classification is very important in the medical field to generate statistics about the causes of illness and causes of death. Many varieties of deep learning algorithms are utilized in different applications as the nature of the data determines which deep learning algorithms are used. The most widely used deep learning algorithms are as follows:

#### 3.4.1. Convolutional Neural Networks (CNNs)

**Figure 2:**
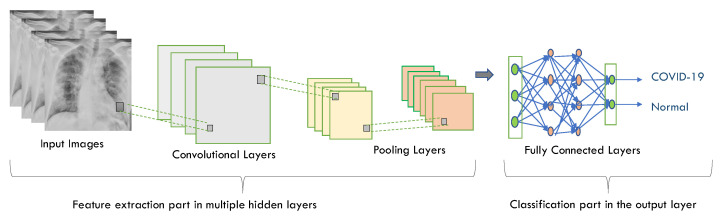
CNN architecture using chest X-ray images as an input.

For image classification, CNN is one of the most commonly used deep neural network types [103]. Unlike neural networks ‘ANN’, where the input is a vector, here the input is a multi-channeled image. CNN operates by extracting features from images directly [104]. The essential features are not pre-trained; they are learned while the network trains on a set of images. This automated feature extraction makes deep learning models more accurate for computer vision tasks such as object classification [105]. CNN learns to detect distinct aspects of an image using many hidden layers. CNN is formed by three main types of layers (convolutional layer, pooling layer, and fully connected layer) [106,107] as shown in Figure 2. The description of these layer types is as follows:

- **Convolutional layer** has a set of filters (or kernels). A kernel or a filter is a collection of weights, where each neuron in one layer is connected to every neuron in the next layer in the neural network using weights. It performs a convolution operation (a linear operation involving a set of weights multiplied (in a dot product mode) by the input is called *convolution*) [108]. To obtain a certain value, the value of dot products are added together;
- **Pooling layer** is applied to the feature maps produced by a convolutional layer. It provides an approach for downsampling feature maps by summarizing the presence of features in patches of the feature map, which leads to reducing the number of parameters and calculations in the network. It recognizes the complex objects in the image and thus preventing overfitting. Average pooling and max pooling are two common pooling algorithms that summarize a feature’s average presence maps;
- **Fully connected layer** connects all of the neurons from the previous layer and assigns each connection a weight. Each output node in the output layer represents a class’s score. Multiple convolutional-pooling layers are merged to generate a deep architecture of nonlinear transformations, which helps to create a hierarchical representation of an image, facilitating the learning of complex relationships.

CNN is widely used in image classification because it is powerful in achieving high accuracy with lowest error rate, but there are some disadvantages as follows: CNN has multiple layers, hence the training process takes a lot of time and also requires a large data set to process and train the neural network [109].

#### 3.4.2. Recurrent Neural Networks (RNNs)

**Figure 3:**
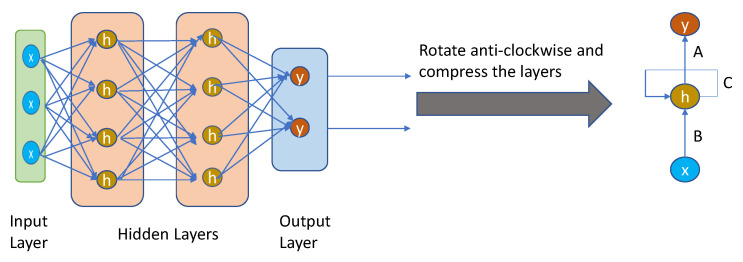
Simple recurrent neural network architecture.

RNNs are widely employed in image labels, speech recognition, time series analysis, machine translation, and natural language processing (NLP). RNNs use some types of feedback, in which the output is fed back into the input as shown in Figure 3.

It is a loop that passes data back to the network from the output to the input. The nodes in different layers of the neural network are compressed to form a single layer of recurrent neural networks. ‘x’ is the input layer, ‘h’ is the hidden layer, and ‘y’ is the output layer. A, B, and C are the network parameters used to improve the output of the model. At any given time t, the current input is a combination of input at x(t) and x(t − 1). The output at any given time is fetched back to the network to improve on the output. The previous elements of a sequence determine the output of the RNNs. Therefore, they are able to remember previous data and use that information in their prediction [110].

RNN is the best example of long-term memory as it remembers all information since it was first used. Using its prior knowledge, it anticipates your other actions. However, there are some drawbacks such as slow computation of this neural network, training can be difficult, and very long sequences cannot be processed if you use relu as an activation function. Therefore, RNN includes less feature compatibility when compared to CNN [111].

#### 3.4.3. Deep Belief Networks (DBNs)

**Figure 4:**
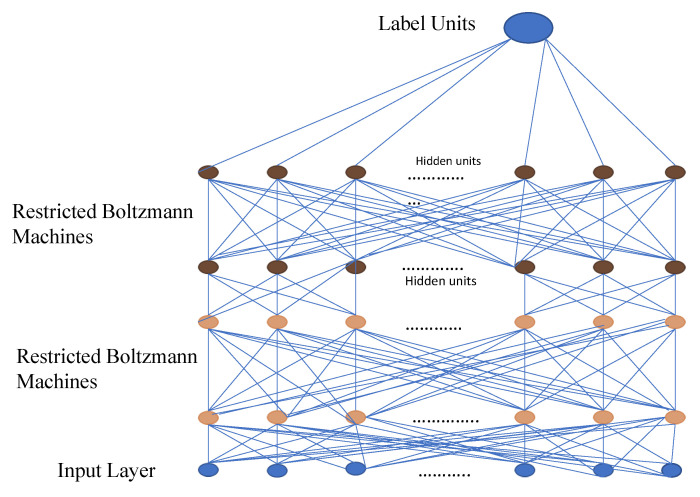
Simple deep belief network architecture.

DBN is a type of deep neural network that comprises a large number of hidden units connected between layers but not between units within each layer as shown in Figure 4.

Restricted Boltzmann Machines (RBMs) are a binary variant of factor analysis. Instead of having multiple factors, the network output will be determined by a binary variable. DBN can be used to extract the in-depth features of the original data. Object recognition, video sequences, and motion capture data are all processed using DBN applications [112,113].

A deep belief network is especially useful when limited training data are available. DBN has specific robustness in classification (size, position, color, and viewing angle rotation). The same neural network approach in a DBN can be implemented on various applications and data types. However, there are some drawbacks including that it requires huge data to perform better techniques such as CNN model, has hardware requirements, and requires classifiers to understand the output [114].

#### 3.4.4. Multilayer Perceptron (MLP)

**Figure 5:**
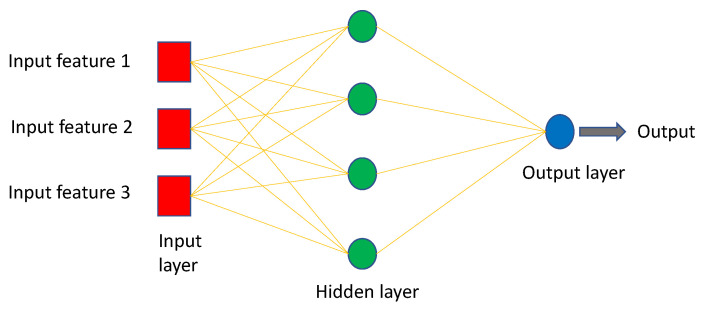
Simple MLP architecture.

MLP is a sort of feedforward neural network made up of multiple layers of perceptrons with activation functions and is a fully connected class of Artificial Neural Network (ANN), where ANN refers to models of human neural networks that are designed to help computers learn. It consists of a large number of highly interconnected processing elements called neurons, and one or more hidden layers. MLP are made up of at least three fully connected layers: input, hidden, and output layers as shown in Figure 5.

MLP might have several hidden layers, and they are employed in applications of machine translation software, complex signal processing, speech recognition, and image recognition [115].

The MLP model is one of the best and simplest types of artificial neural networks, and it works well with both small and large input data. However, one of its drawbacks is that the calculation process is difficult and takes a long time [116].

### 3.5. Ways to Train Deep Learning Models

A pre-trained model is one that has been trained on a large dataset to handle a problem similar to the one we are working on. There are three types of training a deep learning model: learning from scratch, transfer learning, and fine-tuning.

**Figure 6:**
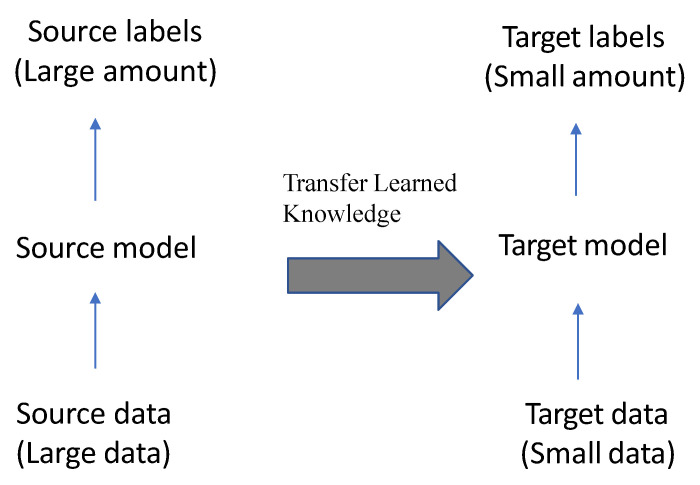
The concept of transfer learning.

- **Learning from scratch** collects a large number of labeled datasets and designs a network architecture to learn the features that may then be used as input to a model (i.e., feature extractor). Feature extraction images may be extracted from a model automatically as in the CNN model or manually using hand-crafted methods such as Histogram of Oriented Gradients (HOG), Intensity Histograms (IH), Scale Invariant Feature Transform (SIFT), Local Binary Patterns (LBP), and Edge Histogram Descriptor (EHD) [117]. For applications with a large number of output classes, this strategy is useful, but it needs more time to train a model [118];
- **Transfer learning** is the process of transferring information from one model to the next, allowing for more accurate model creation with less training data as shown in Figure 6. Instead of starting the learning process from scratch, transfer learning begins with patterns learned while solving a previous problem, allowing for faster progress and improved performance while tackling the second problem [119]. Many studies use transfer learning to enhance their model performance, such as the ones in [94,101,120,121,122];
- **Fine-tuning** is a common technique for transfer learning. In addition, it is making minor changes in order to obtain the desired result or performance, using the weights of a pre-trained neural network model as initialization for a new model trained on the same domain’s data. Except for the output layer, the target model duplicates all model designs and their parameters from the source model and fine-tunes them based on the target dataset. The target model’s output layer, on the other hand, must be trained from scratch. Fine-tuning deep learning involves using weights of a previous deep learning algorithm for programming another similar deep learning process as in [32,123,124]. Because it already has crucial knowledge from a previous deep learning algorithm, its procedure dramatically reduces the time required to develop and process a new deep learning algorithm. When the amount of data available for the new deep learning model is limited, fine-tuning deep learning models can be used, but only if the datasets of the current model and the new deep learning model are similar [125].

### 3.6. Ensemble Learning

Ensemble learning is the process of strategically generating and combining several models, such as classifiers to solve a specific problem [126]. It is largely used to improve a model’s performance (classification, prediction, function approximation, etc.) or to lower the chance of a poor model selection. It can also be used to assign a confidence level to the model’s decision, data fusion, incremental learning, non-stationary learning, pick optimal (or near-optimal) features, and error-correcting. Classifiers may be Support Vector Machine (SVM), SoftMax, Decision Trees, or Naïve Bayes Classifiers. Voting scheme [127,128], bagging [129], boosting [130,131], and stacking [132,133] are the most commonly used ensemble learning algorithms.

### 3.7. Pre-Trained Models

As mentioned before, transfer learning is a machine learning method where we reuse a pre-trained model as the starting point for a model on a new task as shown in Figure 6.

The following are many pre-trained models for image classification and segmentation as:

- **Visual Geometry Group (VGG)** is the most familiar model for image classification. It is a standard CNN with multiple layers [134]. The VGG models are VGG-16 and VGG-19, which supports 16 and 19 convolutional layers, respectively, trained on the ImageNet (*ImageNet* is a database with over 14 million images divided into 1000 categories). VGG-16 takes a long time to train compared to other models, and this can be a disadvantage when we are using large datasets. The main feature of this architecture is that it focuses on basic 3 × 3 size kernels rather than a large number of hyper-parameters (*a kernel* is a matrix of weights that are multiplied with the input to improve the output in a preferred manner) in the convolutional layers and the max-pooling layers of 2 × 2 size. Finally, it has two fully connected (FC) layers for output, followed by a Softmax classifier. The VGG’s weight configuration is publicly available and has been utilized as a baseline feature extractor in a variety of other applications and challenges. VGG-19 differs from VGG-16 in that each of the three convolutional blocks has an extra layer [135]. The work in [136] used VGG-16 for the classification of 14 different thoracic diseases and the work in [137] used the same model for COVID-19 detection. The work in [138] used VGG-19 for the detection of tuberculosis and the work in [139] used VGG-19 in the detection of pneumonia;
- **Inception-V3** Szegedy et al. invented a type of CNN in 2014 [140]. Inception v3 is an image recognition model that has been shown to attain greater than 78.1% accuracy on the ImageNet dataset [141]. Inception models are different from typical CNNs in that they are made up of inception blocks, concatenating the results of many filters on the same input tensor. The model itself is made up of symmetric and asymmetric building blocks, including convolutions, average pooling, max pooling, concatenations, dropouts, and fully connected layers. Batch normalization is used extensively throughout the model and applied to activation inputs. Loss is computed using Softmax. Inception-V3 is a new version of the starting model that was first released in 2015. It has three different filter sizes in a block of parallel convolutional layers (1 × 1, 3 × 3, and 5 × 5). Moreover, a maximum 3 × 3 assembly is performed. The outputs are transmitted to the next unit in a consecutive order. It accepts an entry image size of 299 × 299 pixels [142]. In [119], the authors used this model for the detection of lung nodule disease;
- **ResNet50** is a type of deep neural network that is a subclass of CNNs and is used to classify images. ResNet50 is a variant of ResNet model which has 48 Convolution layers along with one MaxPool and one Average Pool layer [143]. The usage of residual layers to create a new in-network architecture is a major innovation. ResNet50 is comprised of five convolution blocks, each having three layers of convolution. ResNet50 is a residual network that accepts photos with a resolution of 224 × 224 pixels and has 50 residual networks [144]. The work in [120,145] used this model in the classification of 14 different thoracic diseases;
- **Inception-ResNet-V2** is an ImageNet-trained CNN. The network is 164 layers deep and can classify images into 1000 object categories [141]. It is a hybrid approach that combines the structure of inception with the residual connection. It accepts 299 × 299 pixel images and generates a list of estimated class probabilities. The conversion of inception modules into residual inception blocks, the addition of more inception modules, and the creation of a new type of inception module (Inception-A) following the Stem module are among the advantages of Inception-Resnet-V2 [146];
- **DenseNet201** is a 201-layer CNN that receives a 224 × 224 pixel input image. DenseNet201 is a ResNet upgrade that adds dense layer connections. It connects one layer to the next in a feed-forward approach. DensNet201 has direct connections L(L+1)/2 while the standard convolutional networks have *L* layers and *L* connections. In DenseNet, each layer obtains additional inputs from all preceding layers and passes on its own feature-maps to all subsequent layers. Concatenation is used. Each layer is receiving a “collective knowledge” from all preceding layers. Since each layer in DenseNet receives all preceding layers as input, it has more diversified features and tends to have richer patterns [147]. By increasing the amount of computing required, encouraging feature reuse, minimizing the number of parameters, and reinforcing feature propagation, DenseNet can enhance the model’s performance [148];
- **MobileNet-V2** is an improved version of MobileNet-V1 that uses the ImageNet database to train. It contains only 54 layers and a 224 × 224 pixel input image. MobileNetV2 contains the initial fully convolution layer with 32 filters, followed by 19 residual bottleneck layers [149]. Its key distinctive feature is that it uses depth-wise separable convolutions instead of a single 2D convolution. That is, two 1D convolutions with two kernels are used. As a result, training takes up less memory and requires fewer parameters, resulting in a tiny and efficient model. A residual block with a stride of 1 and a downsizing block with a stride of 2 are the two types of blocks. Each block has three layers: a 1 × 1 convolution with ReLU6, a depthwise 3 × 3 convolution with ReLU6, and another 1 × 1 convolution with nonlinearity. MobileNetV2 is a mobile-oriented model that can be used to solve a variety of visual identification tasks (classification, segmentation, or detection) [150]. The work in [151] used MobileNet-v2 in the classification of 14 different thoracic diseases, and the work in [101] used this model for the detection of tuberculosis disease;
- **Xception** is a CNN that has 71 layers called Xception and presented by Chollet [152]. It features depthwise separable convolutions and is a more advanced version of Inception’s architecture. The traditional Inception modules are replaced by depthwise separable convolutions in Xception. It outperforms VGG16, ResNet, and Inception in conventional classification issues when compared to them. It uses a 299 × 299 pixel input image [152];
- **NASNet** is a type of convolutional neural network discovered through a search for neural architecture. It has been trained on over a million images from ImageNet. For a wide variety of images, the network learned rich feature representations. Normal and reduced cells are the basic building blocks [153]. The network accepts 331 × 331 pixel images as input [154]. The work in [135] used this model in lung cancer detection;
- **U-Net** is used for semantic segmentation. It is a convolutional network architecture for fast and precise segmentation of images. It is used for biomedical image segmentation [155]. In the U-Net model, the input images go through several stages of convolutional and pooling, which reduce the height and width of the image as the depth grows after each convolution in down-sampling, followed by fully convolutional and several stages of up-sampling to produce the image mask [156]. The segmentation image size of 512×512 pixel [157,158]. In [159], the authors used this model for segmentation of thoracic fracture disease, and in [100], the authors used U-Net in segmentation of cardiomegaly disease.

### 3.8. Evaluation Criteria

**Figure 7:**
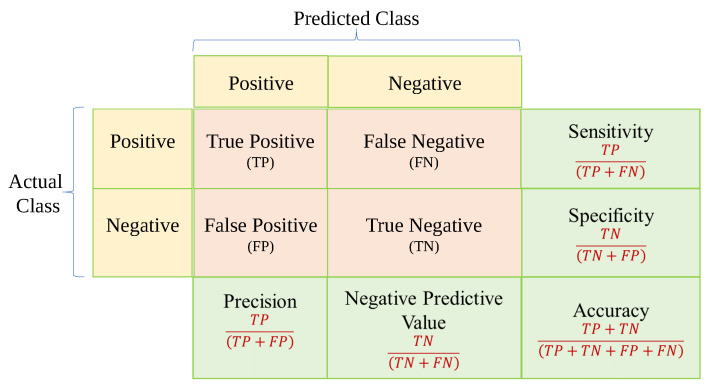
Confusion matrix.

The final step is using a loss function or confusion matrix $C_{ij}$ to determine the number of observations that were categorized properly or wrongly. The loss function is the difference between the expected outcome and the expected output. From the loss function, we can derive the gradients which are used to update the weights. For a data point Yi and its predicted value Yj, where n is the total number of data points in the dataset, the mean squared error (MSE) is defined as in Equation (7). The observed *i* and projected *j* outcome values are compared as shown in Figure 7. The confusion matrix shows the number of correct and incorrect predictions categorized by type of outcome [160]. Recall, Precision, Specificity, Accuracy, Area Under the Curve (AUC), and Receiver Operating Characteristics (ROC) curve can be measured using a confusion matrix. The benchmark metrics are:

(1) $$\begin{array}{c} Accuracy=\frac{TP+TN}{TP+TN+FP+FN} \end{array}$$

(2) $$\begin{array}{c} Precision=\frac{TP}{TP+FP} \end{array}$$

(3) $$\begin{array}{c} Recall=\frac{TP}{TP+FN} \end{array}$$

(4) $$\begin{array}{c} F1-Score=2\times \frac{Precision\times Recall}{Precision+Recall} \end{array}$$

(5) $$\begin{array}{c} Sensitivity=\frac{TP}{TP+FN} \end{array}$$

(6) $$\begin{array}{c} Specificity=\frac{TN}{TN+TP} \end{array}$$

(7) $$\begin{array}{c} MSE=\frac{1}{n}\times \sum_{i=1}^{n}{\left(Y_{i}-Y_{j}\right)}^{2}\; \end{array}$$

### 3.9. Type of Disease

**Figure 8:**
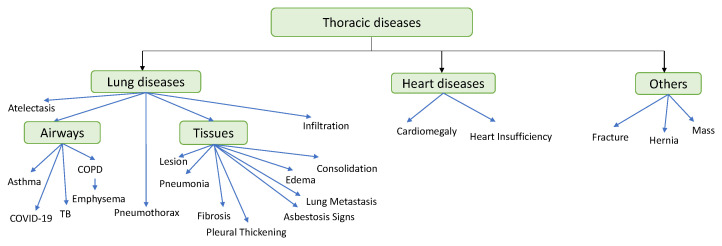
Thoracic diseases classification.

In this paper, we focus on classifying the thoracic diseases into three classes: lung diseases or respiratory system diseases, heart diseases, and others as shown in Figure 8. For each class, we discuss the research that classifies these diseases whether the classification is binary or multiple, the type of image for each disease, the type of AI model that is used to detect this disease, the dataset used, and the performance of each model. *Lung diseases* include pneumonia [139], COVID-19 [137,161,162,163,164,165], edema [166], lesion [135], asbestosis signs [167], consolidation [168], atelectasis [169], COPD [170], pleural thickening [171], fibrosis [172], asthma [173], lung metastasis [98], pneumothorax [174], emphysema [175], tuberculosis (TB) [176], and infiltration [177]. *Heart diseases* include cardiomegaly [128] and heart insufficiency disease [178]. *Other diseases* include fracture [159], hernia [179], and mass [180].

#### 3.9.1. Lung Diseases

These chest diseases affect the structure of the lung tissue, airways, or any part of the respiratory system, causing it to become scarred or inflamed, which makes the lungs unable to fully expand [181]. These diseases appear as opacities on chest radiograph such as pneumonia, MERS-CoV, edema, and consolidation [182]. Table 3, Table 4 and Table 5 summarize the studies on using deep learning to diagnose lung diseases.

##### Lung Diseases That Affect Tissues

Table 3 includes the following diseases: Pneumonia, Fibrosis, Lesion, Pleural Thickening, Asbestosis Signs, Edema, Lung Metastasis, and Consolidation.

**Table 3:** Lung diseases detection summary that affect tissues.

Ref. (Year)	Name of Disease	Input Image Type	Dataset Used	Data Preparation Type	Model Type	Ensemble Technique	Target	Results	Open Issues
[127] (2019)	Pneumonia	X-ray	RSNA Pneumonia Detection Challenge dataset	Data Augmentation including flipping, rotation, brightness, gamma transforms, random gaussian noise, and blur.	RetinaNet and Mask R-CNN	voting scheme	Localization and Classification	Recall 79.3%	A lateral chest X-ray or/and CT images should be augmented to the chest X-ray.Metadata such as age, gender, and view position can be useful in later investigations.
[94] (2021)	Pneumonia	X-ray	Covid Chest X-ray and optical coherence tomography datasets.	intensity normalization. Contrast Limited Adaptive Histogram Equalization (CLAHE).Data Augmentation.	CNN pre-trained on Inception-V3, VGG16, VGG19, DenseNet201, Inception-ResNet-V2, Resnet50, MobileNet-V2, and Xception		Detection and Classification	Accuracy 96.61%, Sensitivity 94.92%, F1-Score 96.67%, Specificity 98.43%, Precision 98.49%	Create a complete system that can detect, segment, and classify pneumonia. Furthermore, performance could be improved by using larger datasets and more advanced feature extraction techniques including color, texture, and shape.
[139] (2021)	Pneumonia	X-ray	ChestX-ray8	image resizing	VGG19	Voting Classifier	Classification and Detection	Accuracy 97.94%	Using texture and shape feature extraction techniques to improve the handcrafted feature vector. Using a suitable classifier system to replace the SoftMax layer. To improve classification accuracy, the fully-connected layer and the drop out layer were modified.
[166] (2020)	Edema	X-ray	MIMIC-CXR	Data Augmentation including translation and rotation	BERT model		Classification and Prediction of the Edema severity level	overall accuracy 89%	Suggest utilizing text to semantically explain the image model.
[183] (2019)	Edema	X-ray	MIMIC-CXR	Data Augmentation including rotation, transformation, and cropping.	Bayesian		predicting pulmonary edema severity	RMS 0.66	To improve the pulmonary edema severity prediction accuracy, researchers suggest using an alternative machine learning approach.
[184] (2018)	Fibrosis	Histopathology	cardiac histological images dataset	Data Augmentation including rotation, flipping, warping, and transformation.	CNN		Segmentation and Detection	Mean DSC is 0.947	Learning data should include proportions of each class and color variations in particular structures, as well as an approximate representation of the attributes in the whole image collection.
[172] (2019)	Fibrosis	CT	LTRC-DB, MD-ILD, INSEL-DB		CNN		Segmentation, Classification, and Diagnosis	Accuracy 81% and F1-score 80%	Use Histopathology or X-ray in the diagnosis.
[168] (2020)	Consolidation	X-ray	Pediatric Chest X-ray	Data Augmentation including cropping, Histogram matching transformation, and Contrast Limited Adaptive Histogram Equalization (CLAHE)	DCNN		Detection and Perturbation visualization (Heatmap)	Accuracy 94.67%	Test DCNN model in multi-classification.
[185] (2021)	Consolidation and (Pneumonia, SARS-CoV-2)	X-ray	COVIDx Dataset	Data Augmentation including flipping, rotation, and scaling.	CNN pre-trained on VGG-19		Classification and Visualization (GradCam)	Accuracy 89.58% for binary classification and 64.58% for multi-classification	Enhance accuracy by using a large amount of data in multi-classification.
[135] (2019)	Lung Lesion/Lung Cancer	CT	LIDC-IDRI		DCNN pre-trained on VGG-19, VGG-16, ResNet50, DenseNet121, MobileNet, Xception, and NASNet		Segmentation and Classification	DenseNet: Accuracy 87.88%, Sensitivity is 80.93%, Specificity is 92.38%, Precision is 87.88%, and AUC is 93.79%. Xception: Accuracy 87.03%, Sensitivity 82.73%, Specificity 89.92%, Precision 84.97%, and AUC 93.24%.	Focusing on the application of deep learning models to small datasets. Using CNNs to synthesize artificial datasets, such as generative adversarial networks.
[186] (2020)	Lung Nodule	CT	Japanese Society of Radiological Technology database	Data Augmentation including horizontal flipping and angle rotation.	CNN		nodule enhancement, nodule segmentation, and nodule detection.	Sensitivity 91.4%	To improve CAD performance, the ROI image can be transformed to an RGB image and combined with additional nodule enhancement images.
[119] (2019)	Lung Nodules	CT	JSRT	Data Augmentation including rotation, flip, and shift.	DCNN pre-trained on Inception-v3		Classification	sensitivity 95.41%, specificity 80.09%	Using ensemble learning to overcome the problem of the deep learning model’s large gap between specificity and sensitivity.
[167] (2021)	Asbestosis Sign	CT	Private dataset		LRCN		Classification and Visualization	Accuracy 83.3%	The LRCN model can be used to diagnose a wide range of lung diseases.
[187] (2022)	Asbestosis	CT	Private dataset	Data Augmentation including zoom, flipping, rotation, and shift. Random sampling.	LRCN (CNN and RNN)		Segmentation and Diagnosis	Sensitivity 96.2%, Specificity 97.5%, Accuracy 97%, AUROC of 96.8%, and F1 score 96.1%	To supplement the limitations of a short dataset, more data should be obtained, and external validation should be done through a multicenter study involving additional hospitals.
[171] (2018)	Pleural Thickening and another 13 different diseases	X-ray	ChestX-ray14	Data Augmentation including cropping and flipping	AG-CNN		Localization and classification	AUC 86.8%	Look into a more accurate localization of the lesion areas. Take on the challenges of sample collecting and annotation (with the help of a semi-supervised learning system).
[145] (2021)	Pleural Thickening and another 13 different diseases	X-ray	ChestX-ray14 and CheXpert		CNN pre-trained on ResNet50		Localization and classification	AUC (Pleural Thickening) 79% of ChestX-ray14 & average AUC 83.5%	Invite a group of top radiologists to work on mask level annotation for the NIH and CheXpert datasets.
[98] (2021)	Lung Metastasis—Lung Cancerv	CT	SPIE-AAPM Lung CT Challenge Data Set	Data Augmentation using GAN network	CNN pre-trained on AlexNet		Classification	Accuracy 99.86%	Adjusting the parameters of each layer to obtain the best parameter combination or implement the optimizer in different network architectures.
[121] (2021)	Lung Cancer	CT	LIDC-IDRI		CNN pre-trained on GoogleNet		Classification	Accuracy 94.53%, Specificity 99.06%, Sensitivity 65.67%, and AUC 86.84%	To increase the classification accuracy of lung lesions in CT images, more study on the GoogleNet network is required.

##### Pneumonia

Pneumonia is an infection that causes breathing difficulties by inflaming the air sacs in one or both lungs.

Using the current deep learning architectures (VGG-16, VGG-19, ResNet-50, DenseNet-201, Inception-ResNet-V2, Inception-V3, MobileNet-V2, and Xception models) for transfer learning to compare current deep CNN architectures and retraining of a baseline CNN, Idri et al. [94] established the best performing architecture for 2-class categorization (pneumonia and normal) based on X-ray images. The OCT and COVID Chest X-ray were the two datasets used. As a result, they determined that the fine-tuned version of Resnet50 operates exceptionally well, with rapid increases in training and testing accuracy (more than 96%). Using transfer learning of current deep learning architectures, they established the best performing architecture for 2-class categorization (pneumonia and normal) based on X-ray images. Dey et al. [139] presented a Deep-Learning System (DLS) to diagnose lung diseases based on X-ray images. The suggested study makes use of traditional chest radiographs as well as chest radiographs that have been processed with a threshold filter. Standard DL models with a SoftMax classifier are utilized for the first experimental evaluation using the ChestX-ray8 dataset, including AlexNet, VGG16, VGG19, and ResNet50. The results showed that VGG-19 has a higher classification accuracy of 86.97% when compared to other approaches. They then used the Ensemble Feature Scheme to modify the VGG19 network to identify pneumonia. VGG19 with an RF classifier has a higher accuracy of 95.70%. When the same experiment was conducted with chest radiographs that had been handled with a threshold filter, the classification accuracy of the VGG19 using the RF classifier was 97.94%.

An automated model for detecting and localizing pneumonia on chest X-ray images were provided by Sirazitdinov et al. [127]. For pneumonia identification and localization, they suggest an ensemble of two convolutional neural networks, Mask R-CNN and RetinaNet, where RetinaNet is a one-stage object detection model that utilizes a focal loss function to address class imbalance during training. The RetinaNet backbone uses ResNet and Feature Pyramid Net (FPN) structures. Based on the FPN structure, a top-down path and horizontal connection are added. Each level of the FPN is connected to the fully convolutional networks, which include two independent subnets that are used for classification and regression. The Mask R-CNN is a Convolutional Neural Network and state-of-the-art in terms of image segmentation. This variant of a Deep Neural Network detects objects in an image and generates a high-quality segmentation mask for each instance.

For the detection of pneumonia, the Faster R-CNN-based technique was used. They used the Kaggle Pneumonia Detection Challenge dataset, which contains 26,684 X-ray images of pneumonia. The recall score was 79.3%.

##### Fibrosis

The pulmonary fibrosis disease is characterized by scarred and damaged lung tissue. These thick, rough tissues make it difficult for your lungs to function properly, and as pulmonary fibrosis worsens, you will start to feel short of breath.

Christe et al. [172] presented a CNN model for the classification and diagnosis of pulmonary fibrosis disease by using CT images. They used three datasets: Lung Tissue Research Consortium Database (LTRC-DB), the Multimedia Database of Interstitial Lung Diseases (MD-ILD), and the Inselspital Interstitial Lung Diseases Database (INSEL-DB). They used the random forest (RF) classifier that was able to recommend a radiological diagnosis. The output accuracy is 81%, and the F1-score is 80%.

Fu et al. [184] developed and tested an elegant convolutional neural network (CNN) for histological image segmentation, particularly those containing Masson’s trichrome stain. There are 11 convolutional layers in the network. The CNN model was trained and tested on a 72-image dataset of cardiac histology pictures (labeled fibrosis, myocytes, and background). The segmentation performance of the model was excellent, with a test mean dice similarity coefficient (DSC) of 0.947.

##### Lesion

Pulmonary lesions, pulmonary nodules, lung nodules, pulmonary nodules, white spots, and lesions are various words for the same thing: an abnormality in the lungs. They are distinct, well-defined spherical opacities with a diameter of less than or equal to 3 cm (1.5 in) that are entirely surrounded by lung tissue, do not touch the lung root or mediastinum, and are not associated with enlarged lymph nodes, collapsed lung, or pleural effusion. A pulmonary nodule might be malignant or benign.

Zhang et al. [135] presented a DCNN model pretrained on VGG-19, VGG-16, ResNet50, DenseNet121, MobileNet, Xception, and NASNet. They showed that DenseNet121 and Xception achieved better results for lung nodule diagnosis. They used the CT Lung Image Database Consortium and the image database resource initiative (LIDC-IDRI). The output accuracy for the DenseNet model is 87.77%, sensitivity is 80.93%, specificity is 92.38%, precision is 87.88%, and AUC is 93.79%. Xception output performance is as follows: 87.03% accuracy, 82.73% sensitivity, 89.92% specificity, 84.97% precision, and 93.24% AUC.

Chen et al. [186] introduced a faster region convolutional neural network (Faster R-CNN) that has been effectively used for computed tomography nodule candidate detection. Before doing nodule detection, they did nodular enhancement and segmentation. They used the database of the Japanese Society of Radiological Technology. The model performed well, with a sensitivity of 91.4% and 97.1%, respectively, with 2.0 and 5.0 false positives per image (FPs/image).

To categorize pulmonary images, Wang et al. [119] employed a DCNN model pre-trained on Inception-v3 to create a viable and practicable computer-aided diagnostic model. The computer-aided diagnostic approach could help clinicians diagnose thoracic disorders more accurately and quickly. They employed the fine-tuned Inception-v3 model based on transfer learning and a variety of classifiers (Softmax, Logistic, and SVM). They worked using the JSRT dataset. The sensitivity of the model was 95.41%, and the specificity was 80.09%.

##### Pleural Thickening

Pleurisy is a disease that causes thickening of the lung lining, or pleura that may cause chest pain and difficulty breathing.

Guan et al. [171] proposed an attention guided convolution neural (AG-CNN) network that avoids noise and improves alignment by learning from disease-specific regions. AG-CNN is divided into three branches. Five convolutional blocks with batch normalisation and ReLU make up the global and local branches. A max pooling layer, a fully connected (FC) layer, and a sigmoid layer are then connected to each of them. Unlike the global branch, the local branch’s input is a local lesion patch that is cropped by the mask formed by the global branch. The fusion branch is then created by concatenating the maximum pooling layers of these two branches. They initially learn about a global CNN branch by looking at global visuals. Then, they used the attention heat map obtained by the global branch to infer a mask to crop a discriminative region from the image.

The ChestX-ray14 dataset was used to train and test the model. The AUC for AG-CNN is 86.8% on average. The average AUC was 87.1% when DenseNet-121 is utilized.

For clinical applications, solving the problem of abnormality localization in addition to categorising abnormalities, further training of these models to locate abnormalities could be employed to address this problem. However, doing so accurately will necessitate a significant number of clinical expert disease localisation annotations.

Ouyang et al. [145] employed a hierarchical attention mining framework that unites activation and gradient-based visual attention in a holistic manner, as well as an attention-driven weakly supervised algorithm. The three layers of attention mechanisms in the hierarchical attention mining framework are foreground attention, positive attention, and abnormal attention. ChestX-ray14 and CheXpert datasets are used in their investigation. The average AUC for the ChetX-ray dataset is 83.5%. The AUC of ResNet50 and ResNet152 increased to 88.8% and 89.5%, respectively, when transfer learning was used.

##### Asbestosis Signs

Asbestosis is a long-term lung illness caused by inhaling asbestos fibers. Long-term exposure to these fibers causes lung tissue scarring and shortness of breath. The disease’s symptoms can range from minor to severe, and they normally do not show up for several years following persistent exposure.

Using medical CT data, Myong et al. [167] presented a Long-term Recurrent Convolution Networks (LRCN) model capable of recognizing the existence and severity of asbestosis. The CNN and RNN models are combined in the LRCN model. LRCN processes the variable-length visual input with a CNN. In addition, their outputs are fed into a stack of recurrent sequence models, which is long short-term memory (LSTM). The final output from the sequence models is a variable-length prediction. DenseNet161 is used to train the CNN model (transfer learning). They used private data from 469 patients who had been screened for asbestosis at Seoul St. Mary’s Hospital in Korea. The purpose of this study was to employ LSTM which is a special type of RNN to address the image classification problem with CT data. The model achieved an accuracy of 83.3%, with a true positive of 81.578% and a true negative of 86%. Additionally, a model was built that can test validity by assisting an expert with a Grad-CAM that can see the judgement.

A lung segmentation and deep learning model-based approach for recognizing patients with asbestosis in segmented computed tomography (CT) images has been developed by Kim et al. [187], which could be used as a clinical decision support system (CDSS). They also suggested that the LRCN model to categorize lungs into normal and asbestosis lungs (CNN extracts image features, and RNN learns the extracted sequence information). They used a private dataset at Seoul St. Mary’s Hospital, which is part of the Catholic University of Korea’s College of Medicine (IRB no. KC17ENSI0379). There were a total of 447 patients, with 275 being healthy and 172 having asbestosis. In addition, 87 of the 172 patients with asbestosis were diagnosed in the early stages, while 85 were discovered in the advanced stages. The algorithm built with the DenseNet201 pre-trained model performed exceptionally well, with a sensitivity of 96.2%, specificity of 97.5%, accuracy of 97%, AUROC of 96.8%, and F1 score of 96.1%.

##### Pulmonary Edema

Excess fluid in the lungs causes this disorder. This fluid gathers in the lungs’ many air sacs, making breathing harder.

Chauhan et al. [166] used the Medical Information Mart for Intensive Care CXR dataset (MIMIC-CXR) to present a Bidirectional Encoder Representations from a Transformers (BERT) neural network model that learns from images and text to assess pulmonary edema severity from chest radiographs, where BERT is a deep learning model in which every output element is connected to every input element, and the weightings between them are dynamically calculated based upon their connection. Overall, the accuracy is 89%.

Liao et al. [183] also measure the severity level of pulmonary edema in CXR images, but by using a Bayesian model for training and testing on the MIMIC-CXR dataset. The root mean squared (RMS) error is 0.66, and the Pearson correlation coefficient (CC) is 0.52.

##### Lung Metastasis

Metastasis of the Lungs or Metastatic Lung Disease Cancer is a malignant tumor that develops elsewhere and spreads to the lungs through the circulation. Breast cancer, colon cancer, prostate cancer, sarcoma, bladder cancer, neuroblastoma, and Wilm’s tumor are all common malignancies that metastasize to the lungs. Any malignancy, on the other hand, has the potential to move to the lungs.

To overcome the difficulty of sparse data, the Generative Adversarial Network (GAN) was presented by Lin et al. [98] to generate computed tomography images of lung cancer. GAN is applied to generate new data automatically. It trains the generator and discriminator networks simultaneously. The former generates new images, and the latter learns to distinguish the fake images from the input of real and generated data. The AlexNet model is applied for the classification of lung cancer into benign or malignant tumors. They used the SPIE-AAPM Lung CT Challenge Data Set that contains 22,489 lung CT images, with 11,407 images of malignant tumors and 11,082 images of benign tumors. The image size is 512 × 512 pixels. The model achieved an accuracy of 99.86%.

Using CT images from the LIDC-IDRI datasets, Ashharet al. [121] evaluated the performance of five convolutional neural network architectures: ShuffleNet, GoogleNet, SqueezeNet, DenseNet, and MobileNetV2 in categorizing lung tumors into two classes: malignant and benign categories. They proved that GoogleNet has the best performance for CT lung tumor classification with a specificity of 99.06%, an accuracy of 94.53%, sensitivity of 65.67%, and AUC of 86.84%.

##### Consolidation

Pulmonary consolidation is an area of normally compressible lung tissue that occurs when that tissue is filled with fluid instead of air.

To detect consolidation lung illness, Rostami et al. [168] deployed a pre-trained deep convolutional neural network (DCNN) VGG16 and DenseNet121 on ImageNet datasets. The dataset they used was the Pediatric Chest X-ray dataset, which contains two classes, normal and pneumonia/consolidation. The model correctly identified consolidation with a 94.67% accuracy.

A CNN classification model pre-trained on VGG-19 was developed by Bhatt et al. [185] for COVID-19 pulmonary consolidations in chest X-ray detection. They look at binary classification to detect consolidation lung disease, followed by multi-classification predictions (normal, pneumonia, and SARS-CoV-2). They used the COVIDx dataset, which includes 66 COVID-19 among the 16,756 chest radiography images. For binary classification, the accuracy was 89.58%, while for multi-classification, it was 64.58%.

##### Lung Diseases That Affect Airways

Table 4 includes the following diseases: Asthma, COPD, TB, and COVID-19.

**Table 4:** Lung diseases detection summary that affect airways.

Ref. (Year)	Name of Disease	Input Image Type	Dataset Used	Data Preparation Type	Model Type	Ensemble Technique	Target	Results	Open Issues
[188] (2021)	COPD	CT	KNUH and JNUH		3D-CNN		Extraction, visualization, and classification	Accuracy 89.3% and Sensitivity 88.3%	Apply a 3D-model using a wide range of datasets.
[170] (2021)	COPD	CT	RFAI	data augmentation processes: random rotation, random translation, random Gaussian blur, and subsampling	MV-DCNN		Classification	Accuracy 97.7%	Applied MC-DCNN to diagnose a variety of lung diseases.
[175] (2019)	Emphysema	CT	private dataset		DCNN		Classification and Detection	Accuracy 92.68%	Use transfer learning to achieve high accuracy.
[189] (2019)	Emphysema and another different 13 diseases	X-ray	ChestX-ray14		CNN		Classification	overall Accuracy 89.77%	Ensemble approaches were used to improve the model’s performance.
[173] (2018)	Asthma	Reports only	Private dataset		DNN		Diagnosis	Accuracy 98%	Apply a different classifier to outperform the DNN algorithm in terms of accuracy.
[190] (2018)	Asthma	Reports only	Private dataset		Bayesian Logistic Regression		Prediction for Asthma disease	Accuracy 86.3673%, Sensitivity 87.25%	Check if there is an increase in the accuracy when including more patients in the dataset, using the posteriors from this study as priors for the new dataset.
[137] (2020)	COVID-19	CT	Private dataset	Histogram equalization features extraction Intensity transformation	CNN pre-trained on VGG-16		Classification	Precision 92%, Sensitivity 90%, Specificity 91%, F1-Score 91%, Accuracy 90%	It is possible to use deep learning networks with more complex backbone architecture.GANs can be developed to increase the number of suitable images for network training and hence improve the model’s performance.
[131] (2020)	COVID-19	CT	Private dataset	Volume features based on segmented infected lung regions, Histogram distribution, Radiomics features.	CNN	Boosting	Segmentation and Classification	Accuracy 91.79%, Sensitivity 93.05%, Specificity 89.95%, AUC 96.35%, Precision 93.10%, and F1-score 93.07%	Plan to collect more data from patients with different diseases and apply the AFSDF approach to further COVID-19 classification tasks (e.g., COVID-19 vs. Influenza-A viral pneumonia and CAP, severe patients vs. non-severe patients).
[122] (2021)	COVID-19, Pneumonia, Tuberculosis	X-ray	Pediatric CXRs, IEEE COVID-19 CXRs, and Shenzhen datasets.	Data Augmentation including rotation, shift, and adding noise.	DenResCov-19		Classification	Precision 82.90%, AUC 95%, and F1-Score 75.75%	Increase the number of classes to address more lung illnesses. The number of COVID-19 patients should be raised.
[191] (2021)	COVID-19	X-ray	RSNA dataset	Data Augmentation including zoom, flipping, rotation, translation, and shift.	COVID-Net CXR-S		Detection and Classification	Sensitivity (level1) 92.3%, Sensitivity (level2) 92.85%, PPV (level1) 87.27%, PPV (level2) 95.78%, Accuracy 92.66%	Build innovative clinical decision support technologies to aid clinicians all throughout the world in dealing with the pandemic.
[133] (2022)	COVID-19	X-ray	COVID-19 dataset, chest-X-ray, COVID-19 pneumonia dataset, private dataset collected from MGM Medical College and hospital		DCNN	Stacking	Classification and Detection	Accuracy 88.98% for three classifications and 98.58% for binary classification.	Using more public datasets will improve the model’s accuracy.
[192] (2022)	COVID-19	CT	COVID-19 CT Images Segmentation	segmentation	DRL		Image segmentation	Precision 97.12%, a sensitivity of 79.97%, and a specificity of 99.48%	The mask extraction stage could be improved. In addition, more complex algorithms, approaches, and datasets appear promising to improve system performance.
[193] (2019)	Tuberculosis	Sputum Smear Microscopy	ZNSM-iDB dataset	Data Augmentation including rotation and translation	RCNN pre-trained on VGG-16		localization and classification	Recall 98.3% Precision 82.6% F1-Score 89.7%	Planning to expand the amount of data used in a deep network.
[101] (2020)	Tuberculosis	X-ray	NIAID TB dataset and RSNA dataset	Data Augmentation including cropping.	CNN pre-trained on (Inception-V3, ResNet-18, DenseNet-201, ResNet-50, ResNet-101, ChexNet, SqueezeNet, VGG-19, and MobileNet-V2) and UNet for segmentation.		Lung Segmentation and TB Classification	Without Segmentation: Accuracy 96.47%, Precision 96.62%, and Recall 96.47% With Segmentation: Accuracy 98.6%, Precision 98.57%, and Recall 98.56%	Split lungs into patches that can be fed into a CNN model, perhaps improving performance even more.
[176] (2021)	Tuberculosis	X-ray	Montgomery County (MC) CXR dataset, Shenzhen dataset, RSNA Pneumonia Detection Challenge dataset, Belarus dataset, and COVID-19 radiography database		EfficientNet and Vision Transformer	Boosting	Classification & Detection	Accuracy 97.72%AUC 100%	Planning to add new baselines to compare to the tool that has been developed.Planning to release a mobile app that can run on small devices like smartphones and tablets.
[138] (2021)	Tuberculosis	X-ray	Montgomery County dataset (MC) and Shenzhen dataset (SZ)	Histogram Equalization & Contrast Limited Adaptive Histogram Equalization (CLAHE).	CNN pre-trained on VGG19	Stacking	Segmentation and Detection TB disease	AUC 99.00 ± 0.28/98.00 ± 0.16 for MC/SZ For the MC/SZ accuracy 99.26 ± 0.40/99.22 ± 0.32.	Propose scalability testing for the proposed approach on large datasets. Use big data technologies like distributed processing and/or Map-Reduced based approaches for complex network building and feature extraction.

##### Tuberculosis

Tuberculosis (TB) is a bacterial infection caused by Mycobacterium tuberculosis bacteria. The bacteria most commonly assault the lungs, but they can also harm other regions of the body. When a person with tuberculosis coughs, sneezes, or talks, it spreads via the air.

Tuberculosis was was properly detected from chest X-ray images using data augmentation, image segmentation, and deep-learning classification approaches. Rahman et al. [101] employed nine distinct deep CNNs for transfer learning (ResNet18, ResNet50, ResNet101, ChexNet, InceptionV3, VGG19, DenseNet201, SqueezeNet, and MobileNet). They used the NIAID TB dataset as well as the RSNA dataset. Without segmentation, the output classification accuracy, precision, and recall for tuberculosis detection were 96.47%, 96.62%, and 96.47%, respectively, but with segmentation, they were 98.6%, 98.57%, and 98.56%.

Duong et al. [176] presented a practical method for detecting tuberculosis from chest X-ray pictures. The Montgomery County (MC) CXR, the Shenzhen dataset, the Belarus dataset, the COVID-19 dataset, the COVID-19 Radiography Database, and the RSNA Pneumonia Detection Challenge dataset were all utilized to train and evaluate the Hybrid EfficientNet with a Vision Transformer model. The model achieved a 97.72% accuracy and 100% AUC.

Khatibi et al. [138] introduced a new Multi-Instance Learning (MIL) strategy that combines CNNs, clustering, complex network analysis, and stacked ensemble classifiers for TB diagnosis from CXR images. To detect tuberculosis, they employed a multi-instance classification based on a CNN model.They used two datasets for the TB scans: Shenzhen dataset (SZ) and the Montgomery County dataset (MC). The accuracy of MC/SZ is 99.26 ± 0.40/99.22 ± 0.32, and the AUC is 99.00 ± 0.28/98.00 ± 0.16. Support Vector Machine (SVM), Logistic Regression (LR), Decision Tree (DT), Random Forest (RF), and Adaboost were among the classifiers utilized.

El-Melegy et al. [193] presented a Faster Region-based Convolutional Neural Network (RCNN) to detect Tuberculosis Bacilli using Sputum Smear microscopy images. They employed the ZNSM-iDB public database, which includes auto-focused data, overlapping objects, single or few bacilli, views without bacilli, occluded bacilli, over-stained views with bacilli, and artifacts. The model achieved F1-Score of 89.7%, a recall of 98.3%, and precision of 82.6%.

##### COVID-19

This disease caused by the severe acute respiratory syndrome coronavirus (SARS-COV-2) is called emerging coronavirus disease (COVID-19). COVID-19 appeared in late 2019, and it appears as a ground-glass opacity (GGO) on radiographs. In March 2020, COVID-19 was declared a global pandemic by the WHO.

By using a chest CT scan from Tabriz’s Alinasab Hospital, Sadjadi et al. [137] demonstrated a deep convolutional neural network (DCNN) model for classification of COVID-19 versus healthy individuals, where DCNN is a CNN that consists of several layers using a three-dimensional neural pattern. There were 131 COVID-19 patients and 150 normal cases controls in this study, which employed a total of 10,979 CT images. VGG16 was used to pretrain a CNN model. They scored 92% precision, 90% sensitivity, 91% specificity, 91% F1-Score, and 90% accuracy.

An adaptive feature selection guided deep forest (AFSDF) method was proposed by Sun et al. [131] for COVID-19 classification from chest CT images. This model was built using a high-level representation of the features. A feature selection approach was applied to the trained deep forest model to remove feature redundancy. They used a private dataset that included 1027 individuals with community-acquired pneumonia (CAP) or non COVID-19 and 1495 patients with COVID-19. The model achieved 91.79% accuracy, specificity, sensitivity, and area under the ROC curve, respectively, were 89.95%, 93.05%, and 96.35%.

Mamalakis et al. [122] presented a new deep transfer learning pipeline network (DenResCov-19) based on chest X-ray images to diagnose patients with COVID-19, pneumonia, and tuberculosis. They have added an extra layer with CNN blocks to combine these two models (DenseNet-121 and ResNet-50) and achieve superior performance over either of them. They put their proposed network to the test on classification problems with two classes (pneumonia vs. healthy), three classes (including COVID-19), and four classes (including tuberculosis). In all four datasets, the proposed network was able to correctly classify these lung diseases, and it outperformed the benchmark networks, DenseNet and ResNet. For the four classes, precision is 82.90 percent, AUC is 95 percent, and F1-Score is 75.75%.

COVID-Net CXR-S was introduced by Aboutalebi et al. [191]. It is a CNN model that uses CXR images to predict the severity of a SARS-CoV-2 positive patient’s airways. With customized macroarchitecture and microarchitecture designs for COVID-19 diagnosis from chest X-ray images, the COVID-Net backbone design demonstrates sparse long-range connectivity and a significant architectural diversity. To give better representational capabilities while maintaining minimal architectural and computational difficulties, the network architecture used projection–expansion–projection–expansion (PEPE) patterns, which are light-weight design patterns. The model classifies input images into two levels of severity. They used the RSNA dataset. The model achieved Level 1 sensitivity, Level 2 sensitivity, level 1 Positive Predictive Value (PPV), Level 2 PPV value, and accuracy of 92.3%, 92.85%, 87.27%, 95.78%, and 92.66%, respectively. They proved that a COVID-Net CXR-S model has high performance compared with CheXNet and ResNet-50.

Deb et al. [133] presented a DCNN model to classify COVID-19 disease. They used VGGNet, GoogleNet, DenseNet, and NASNet to pre-train the model. They used two publicly available datasets and one private dataset. They demonstrated that a multi-model ensemble architecture outperforms a single classifier in terms of performance. When using a public dataset, the model achieved an accuracy of 88.98% for three class classifications (COVID-19, Normal, and Community-Acquired Pneumonia (CAP)) and, for binary class classification, they reported an accuracy of 98.58%. The model achieved accuracy of 93.48% when they used private dataset.

In order to extract visual features from COVID-19-infected areas and deliver an accurate clinical diagnosis while optimizing the pathogenic diagnostic test and cutting down on time, Allioui et al. [192] proposed deep reinforcement learning (DRL) mask extraction-based methodologies. DRL used to minimize the long-term manual mask extraction and enhance medical image segmentation frameworks. They used a public CT images dataset. The model achieved a precision of 97.12% with a Dice of 80.81%, a sensitivity of 79.97%, and a specificity of 99.48%.

##### Asthma

It is a disorder in which the airways narrow and swell, producing excess mucus and making breathing difficult, coughing, and shortness of breath.

To diagnose adult asthma, a deep neural network (DNN) was presented by Tomita et al. [173], where DNN is a neural network with some level of complexity, usually at least two layers. They used a private dataset derived from clinical records of 566 adult outpatients who presented to Kindai University Hospital for the first time with non-specific respiratory symptoms. The output accuracy result is 98%.

Spyroglou et al. [190] presented a Bayesian Logistic Regression model to predict asthma. Data were gathered from 147 patients by the Pediatric department of the University Hospital of Alexandroupolis, Greece during the period from 2008 to 2010. The output accuracy for prediction was 86.3673% and the sensitivity of 87.25%.

##### COPD

Chronic Obstructive Pulmonary Disease (COPD) is a set of diseases that cause airflow restrictions in the lungs and breathing difficulties. Emphysema and chronic bronchitis are two conditions that make breathing difficult. The lungs rely on the natural flexibility of the airways and alveoli to remove air from the body. In the case of COPD, the lungs lose their elasticity, which leads to their expansion, which leads to the retention of air inside them [194]. COPD affects millions of people, although it is rarely recognized or treated [195]. Changes in the airways of the lungs are an early sign of COPD. According to WHO estimates, COPD is the third largest cause of mortality worldwide, causing 3.23 million deaths in 2019 [196]. A chest X-ray may not show COPD until it is severe, and the images may show enlarged lungs or airways (bullae), cardiac stenosis, or a flat diaphragm. Thus, doctors may request a computerized tomography (CT) scan after the X-ray scan to obtain a clearer picture to help diagnose them [197].

Using CT scans, Bao et al. [170] presented a 15-direction Multi-View Deep Neural Network (MV-DCNN). To create the MV-DCNN, they used 15 anti-aliased ResNet18 models as well as a classification layer. The three steps of the multi-View DCNN algorithm are as follows: The initial step is to extract images from three-dimensional data from 15 different angles. The second stage is to improve the data in each of these 15 views. To extract and categorize the features, the final step is to build 15 Multi-View DCNN (MV-DCNN) models. They used RFAI’s synthetic texture datasets to test the accuracy of 3D texture feature classification techniques. COPD classification has an output accuracy of 97.7%.

A new 3D-cPRM classification approach for COPD grouping was developed by Ho et al. [188] using a 3D-CNN model and the parametric-response mapping (PRM) method. The researchers then utilized a technique called gradient-weighted class activation mapping (Grad-CAM) to highlight the key components of the CNN learning process. They used data from the Institutional Review Boards of Kangwon National University Hospital (KNUH) and Jeonbuk National University Hospital (JNUH). CT scans at KNU and JNU Hospitals yielded 596 patients (204 with COPD and 392 without COPD). The model had a sensitivity of 88.3% and an accuracy of 89.3%.

##### Emphysema

A symptom of a lung disorder is shortness of breath. In persons with emphysema, the air sacs in the lungs (alveoli) become damaged, and the alveoli’s inner walls weaken and burst over time, resulting in bigger air gaps rather than many smaller ones. The surface area of the lungs is reduced, limiting the amount of oxygen that reaches the bloodstream. Emphysema is a part of COPD.

Peng et al. [175] using multi-scale deep convolutional neural networks, a novel deep learning DCNN technique for pulmonary emphysema classification was presented. The findings revealed that, (1) when compared to a single scale setup, the multi-scale technique was far more effective. (2) In terms of performance, the model exceeded current approaches. (3) The severity of emphysema measured agreed well with various pulmonary function indices. They worked using a private dataset. The accuracy of the classification output is 92.68 percent.

Choudhary et al. [189] presented a CNN model used to predict the probability of one of the fifteen diseases, including emphysema. They used the ChestX-ray14 dataset. An overall accuracy of 89.77% was achieved for the classification of the different diseases.

##### Infiltration

A pulmonary infiltrate is a substance that is denser than air and persists within the parenchyma of the lungs, such as pus, blood, or protein. Tuberculosis is related to pulmonary infiltrates. Table 5 provides a summary of some of the infiltration disease literature.

**Table 5:** Atelectasis, Pneumothorax, and Infiltration diseases detection summary.

Ref. (Year)	Name of Disease	Input Image Type	Dataset Used	Data Preparation Type	Model Type	Ensemble Technique	Target	Results	Open Issues
[169] (2018)	Atelectasis and another 13 different diseases	X-ray	ChestX-ray14		ChestNet		Classification and Visualization	Average AUC 0.7810	Concentrate on understanding the relationships between those illness image descriptors and implementing them into the computer-aided diagnosis procedure.
[151] (2021)	Atelectasis and another 13 different diseases	X-ray	ChestX-ray14	Data Augmentation including rotation, flipping, and transformation	MobileNet V2		Classification and Prediction of chest 14 disease	Average AUC 0.811 and Accuracy above 90%	In the medical field, a light neural network design can be used. Look into using new architectures to take advantage of label dependencies and segmentation data.
[174] (2019)	Pneumothorax	X-ray	ChestX-ray14	Data Augmentation including translating, scaling, rotating, horizontal flipping, windowing, and adding noise	CNN for classification MIL for localization FCN for segmentation	linear combination (Ensemble Averaging)	Classification, localization, and segmentation	AUC (Classification) 96%, AUC (Localization) 93%, and AUC (Segmentation) 92%	Use other techniques to combine the three approaches.
[198] (2019)	Pneumothorax	CT	Private dataset		CNN		Detection and localization	Accuracy 87.3%	To improve the model’s performance, use data from multiple sources.
[177] (2018)	Infiltration and another different 13 diseases	X-ray	ChestX-ray14		CNN for Classification CPNN and BPNN for Diagnosis Chest diseases		Classification & Diagnosis	CNN Accuracy 92.4%BPNN Accuracy 80.04%CPNN Accuracy 89.57%CNN with GIST Accuracy 92%VGG16 Accuracy 86%VGG19 Accuracy 92%	Propose employing several transfer learning strategies to improve model accuracy.
[136] (2019)	Infiltration and another different 13 diseases	X-ray	ChestX-ray14		CNN pre-traind on VGG-16		Classification & Visualization	Accuracy 83.671% (scratch CNN) and 97.81% (transfer learning)	Using a fine-tuning model other than the VGG-16 to analyze medical images can be a viable option.

Abiyev et al. [177] explained the applicability of CNN technology to classify chest X-ray diseases. Backpropagation neural networks (BPNNs) and competitive neural networks (CpNNs) with unsupervised learning are being utilized to diagnose chest diseases. All of the networks were trained and tested using the ChestX-ray14 database. CNN has a 92.4% output performance, BPNN has an 80.04% output performance, and CPNN has an 89.57% output performance.

Hazra et al. [136] presented first a CNN architecture including convolutional, activation, pooling, and fully connected layers, followed by a Softmax layer that delivers the likelihood of the output for each type of sickness. Then, a CNN model was trained using the ChestX-ray14 dataset and a pre-trained VGG-16 model. Using Grad-CAM, they were able to see how the model performed against a test image. They obtained 83.671% accuracy (scratch CNN) and 97.81% accuracy (transfer learning).

##### Atelectasis

When the patient’s lung sacs do not inflate properly, the blood may be unable to supply oxygen to your organs and tissues, resulting in atelectasis. Table 5 provides a summary of some of the atelectasis disease literature.

Wang et al. [169] proposed a ChestNet model for diagnosing chest diseases with X-ray images consists of two branches: attention and classification. The attention branch exploits the correlation between class labels and the locations of pathological abnormalities, allowing the model to focus adaptively on the pathologically abnormal regions. The classification branch (ResNet-152 model) serves as a uniform feature extraction–classification network, freeing users from troublesome handcrafted feature extraction. Six convolutional layers make up the attention branch: 1 × 1, 3 × 3, and 1 × 1 kernels are used in the first three convolutional layers, which are each followed by a ReLU activation function. The ChestX-ray14 dataset was used. ChestNet’s overall AUC is 0.7810, while Atelectasis disease’s AUC is 0.7433.

Abdelbaki et al. [151] presented the MobileNet V2 model (CNN + Additional Neural Network layers) for classifying and predicting frontal thoracic X-ray lung diseases. They used the NIH ChestX-ray14 database. The AUC average of 81.1% has an accuracy of more than 90% and a specificity of 97.3%. Atelectasis has an accuracy of 79.6% and a specificity of 96.8%.

##### Pneumothorax

A pneumothorax or a deflated lung occurs when a collapsed lung causes an abnormal collection of air in the pleural space between the lung and the chest wall. The most common symptoms are dyspnea and severe pain on one side of the chest. A pneumothorax is a complete or partial collapse of the lung that needs to go to medical attention immediately. Table 5 provides a summary of some of the pneumothorax disease literature.

Gooßen et al. [174] compare and contrast three distinct deep learning algorithms for detecting and localizing pneumothorax in chest X-ray images (CNN, multiple-instance learning, and fully convolutional networks). To predict 14 illnesses, a CNN model trained on the ChestX-ray14 dataset and pre-trained on ResNet-50. The dense layer for pathology prediction was replaced by a new binary classification layer for pneumothorax identification. Multiple-Instance Learning (MIL) combines classification and localization while only requiring image-level labels for training. Fully Convolutional Networks (FCNs) are more advanced networks that are designed for semantic segmentation. They combined the separate methods in a linear fashion. The three approaches (CNN, MIL, and FCN) had AUCs of 96%, 93%, and 92%, respectively. The total classification performance was improved by combining the proposed three approaches as an ensemble.

Based on the whole 26-layer you only look once (YOLO) model, a CNN model was proposed by Park et al. [198]. The YOLO model was utilized to determine the lesions’ bounding boxes. The CNN model was developed using a proprietary dataset that included 1596 chest radiographs of pneumothorax patients of varying severity, as well as 11,137 of normal cases, which were gathered from two tertiary referral hospitals. The CNN model performed well in diagnosing pneumothorax on chest radiographs, with an overall accuracy of 87.3%.

#### 3.9.2. Heart Diseases

Cardiovascular or Heart diseases (CVDs) are diseases that impact your heart’s structure or function, such as: cardiomegaly and heart insufficiency diseases [199]. Cardiovascular disease (CVD) is the major cause of death in the world which causes narrowing or blockage of blood vessels, causing shortness of breath and chest pain. According to the World Health Organization (WHO), 17.9 million people died from cardiovascular diseases in 2019, accounting for 32% of all fatalities worldwide [200]. The data in Table 6 illustrate the summary of heart disease detection.

##### Cadiomegaly

Many studies have looked at the detection of cardiomegaly with other abnormalities in a multi-classification situation, predicting all available labels from the datasets provided as in [100,120,128] and some studies detect cardiomegaly in a binary classification as [201].

Ammar et al. [128] presented a cardiac segmentation and diagnosis through an automated pipeline based on a private MRI images dataset of 150 patients from the Dijon Hospital (Medical Image Computing and Computer Assisted Intervention in the Post-2017 Era (MICCAI)). They employed a complete CNN model for classification and UNet deep learning segmentation network. To classify heart diseases, they utilized a multilayer perceptron (MLP), support vector machine (SVM), and a random forest (RF). As a result of this procedure, the accuracy was 92%.

Sogancioglu et al. [100] used the publicly available ChestX-ray14 dataset for Classification to study the detection of cardiomegaly on frontal chest radiographs using two alternative deep-learning approaches: anatomical segmentation and image-level classification. They trained a typical U-net architecture on a separate JSRT dataset to partition the heart and lung areas. They used ResNet18, ResNet50, and DenseNet121 in the classification. The AUC for segmentation is 0.977, while the AUC for classification is 0.941 as a result. They will look into applying the segmentation-based method to other diagnostic procedures.

The same ChestX-ray14 dataset was used to classify the 14 diseases, Nickisch et al. [120] looked at the performance of multiple network architectures including ResNet-38, ResNet-50, and ResNet-101 to classify 14 different diseases. ResNet-50 achieved an elevated AUC of 0.822 on average.

DCNN was used by Candemir et al. [201] to automatically detect cardiomegaly in digital chest X-rays. They used and fine-tuned various deep CNN architectures to detect cardiomegaly disease. Following that, the researchers provided a CXR-based pre-trained model in which they fully trained an architecture (AlexNet, VGG-16, VGG-19, and InceptionV3) with a large CXR dataset. Finally, they investigated the association between the severity of the disease and a Softmax probability of an architecture. The datasets they used were the NLM-Indiana Collection and the NIH-CXR, both of which are freely available. The accuracy of the NIH-CXR dataset is 88.24% (training set: NIH set and 30% of Indiana Collection; test set: 70% of Indiana Collection) and 89.86% (training set: NIH set and 30% of Indiana Collection).

**Table 6:** Heart diseases’ detection summary.

Ref. (Year)	Name of Disease	Input Image Type	Dataset Used	Data Preparation Type	Model Type	Ensemble Technique	Target	Results	Open Issues
[128] (2021)	Heart disease	MRI	Automated Cardiac Diagnosis Challenge (ACDC-2017)	Handcrafted features, data augmentation, and ROI extraction	CNN for Classification and UNet for Segmentation	Voting technique	Classification and diagnosis of Heart disease	Accuracy 92%	Planning to investigate for more enhancement and improvement of the current result.
[100] (2020)	Cardiomegaly	X-ray	ChestX-ray14	Data Augmentation	ResNet18, ResNet50, and DenseNet121 for classification and UNet for Segmentation		Segmentation and classification	AUC for segmentation is 0.977 and AUC for classification is 0.941	Investigate whether the segmentation-based approach may be used for other diagnostic tasks.
[120] (2019)	Cardiomegaly and other 13 diseases	X-ray	ChestX-ray14	Data Augmentation	CNN pre-trained on ResNet50		Classification	AUC average is 0.822	Investigate other model architectures, new architectures for leveraging label dependencies and incorporating segmentation information.
[201] (2018)	Cardiomegaly	X-ray	NIH-CXR and NLM-Indiana datasets		CNN pre-trained on VGG-16, VGG-19, AlexNet, and InceptionV3		Detection	Accuracy is 0.8986	In addition to image-based clues, look into other factors such as lung size, rib-cage measurements, and diaphragm lengths.
[178] (2018)	Heart Failure	Histopathology	Private dataset collected from the Cardiovascular Research Institute	Data Augmentation including cropping, rotation, image mirroring, and stain color	CNN		Classification and detection	Sensitivity 99% and specificity 94%	The ability of CNNs to detect pre-clinical disease must be evaluated. Focus on heart failure prognostic modeling and post-transplant rejection surveillance, as well as etiologic classification of cardiomyopathy etiologies.
[202] (2021)	Heart Failure	reports only	IBM Commercial and Medicare Supplement Databases		LSTM algorithm-based sequential model architecture	Boosting	Detection of Heart Failure and severity classification	AUC 0.861	Better regularization approaches, models pre-trained on other datasets, and the use of larger datasets with more detailed clinical data are all possible options to increase the performance of the model.

##### Heart Failure

Heart failure, or insufficiency, refers to the heart’s inability to properly pump blood throughout the body. This occurs when the heart becomes too weak or stiff. It does not indicate that the heart has stopped working; it only requires some assistance to help it function better.

Nirschl et al. [178] created a CNN classifier to predict clinical heart failure in 209 patients using H&E stained whole-slide images. They used private data from the University of Pennsylvania’s Cardiovascular Research Institute and Department of Pathology and Laboratory Medicine, which they received and analyzed. They proved that the CNN model is able to detect patients with heart failure or severe pathology with a sensitivity of 99% and a specificity of 94%.

To predict hospital admission, exacerbation of HF, at 30 and 90 days in patients with heart failure with low ejection fraction (HFrEF), Wang et al. [202] employed a sequential model architecture based on bidirectional long-term memory (Bi-LSTM) layers. They used two sets of data: the HFrEF patient group, which had only 47,498 patients but had almost two million medical events or interactions obtained from claims, and the general patient group data collection. The AUC is 86.1%.

#### 3.9.3. Others

This class includes diseases that affect bones or muscles of the chest such as fracture, hernia, and mass, as shown in Figure 8. Table 7 illustrates the summary of mass, fracture, and hernia diseases detection.

**Table 7:** Other diseases’ detection summary.

Ref. (Year)	Name of Disease	Input Image Type	Dataset Used	Data Preparation Type	Model Type	Ensemble Technique	Target	Results	Open Issues
[159] (2021)	Fracture	CT	Private		R-CNN for classification & UNet for Segmentation	weighted average of probabilities	Segmentation and detection	Average Sensitivity 89.2% and Precision 88.4%	A 3D-CNN model can be used as a classification model to further classify the observed rib fracture types from the existing model. The performance of the rib segmentation and labelling algorithm must be improved.
[203] (2020)	Fracture	CT	Private		R-CNN pre-trained on ResNet101		Detection and classification	For three multicenter datasets: Precision 80.3% and Sensitivity 62.4% For five multicenter datasets: Precision 91.1% and sensitivity 86.3%	The anatomical location was identified using a three-dimensional deep learning and tracking approach.
[179] (2019)	Hernia and another 13 different diseases	X-ray	ChestX-ray14	Data Augmentation including flipping	CNN pre-trained on DenseNet121		Classification	AUC 84.3% and AUC for Hernia only 96.37%	Entropy weighting loss improved the binary classification of Hernia.
[204] (2021)	Hernia and another 13 different diseases	X-ray	ChestX-ray14		CNN pre-trained on DenseNet121	multiscale ensemble module	Classification	AUC 82.6%	Using pathologically abnormal region annotations to regularize attention learning.Addressing the uncertainty that existed in noisy labels.
[180] (2020)	Mass	X-ray	ChestX-ray14		Quibim App Chest X-ray Classifier		Detection	Sensitivity 76.6%, AUC 91.6%, Accuracy 83%, and Specificity of 88.68%.	During the diagnostic procedure, build the four algorithms that were mentioned in this paper to improve sensitivity and specificity.
[205] (2017)	Mass	X-ray	JSRT dataset		RCNN		detection and localization	Accuracy 53%	Compare the RCNN algorithm to other state-of-the-art mass detection algorithms.

##### Fracture

Chest fractures are injuries to the chest wall, such as the bones, skin, fat, and muscles that protect your lungs or any of the organs inside the chest.

Wu et al. [159] used a three-dimensional rib segmentation model (U-Net) and a deep learning R-CNN pre-trained on the ResNet50 algorithm capable of recognizing rib fractures and related anatomic locations on CT images. First, they scanned the rib fractures and the ribs segmented section by section using a two-dimensional (2D) detection network. To improve rib segmentation accuracy, a three-dimensional (3D) network was used. With an 84.3% free-response receiver operating characteristic (FROC) score on the test set 1, the model correctly diagnosed rib fractures. With a detection sensitivity of 84.9%, a precision of 82.2%, and an F1-score of 83.3%, the system did well in the test set 2. The model achieved an AUC of 93%, a sensitivity of 87.9%, and a specificity of 85.3% on the test set 3. The model received an 82.7% dice score and a 96% accuracy for rib segmentation.

Zhou et al. [203] also demonstrated an R-CNN model that can detect and categorize rib fractures in computed tomography (CT) images and generate structured reports. First, CNN’s raw output was used, and then the merged structured report was used. They used private data from three hospitals. There were 1079 patients in this study. The results indicated that the model does a good job of classifying rib fractures into three classes (old, healing, and fresh fractures). Fresh fractures and healing fractures had higher detection efficiency than old fractures (F1-scores of 84.9%, 85.6%, and 77%, respectively), and the model’s robustness was good in the five multicenter/multiparameter validation sets (all mean F1-score 80%). The five radiologists’ precision climbed from 80.3% to 91.1%, while their sensitivity increased from 62.4% to 86.3%. The radiologists’ diagnosis time was decreased by 73.9 s.

##### Hernia

The section of a lung that pushes through a tear or bulges through a weak area in the chest wall, neck canal, or diaphragm is called a lung hernia.

To increase model performance, an entropy weighting loss was presented by Mo et al. [179] to notice inter-label relationships and make full use of classes with fewer cases than others. They tested out three different deep learning models (VGG16, ResNet50, DenseNet121). Under the Chest X-ray14 dataset, DenseNet121 produced better results, with an AUC score of 84.3% on average.

The triple-attention learning model was presented by Wanget al. [204] for computer-aided diagnosis (CAD) of thoracic diseases. For element-wise, channel-wise, and scale-wise attention learning, the model combines three attention modules into a cohesive framework. It was pre-trained using DenseNet121 for feature extraction. The deep learning model can use element-wise attention to focus on areas with pathological abnormalities, and scale-wise attention to rescale feature maps. The utilized dataset was the ChestX-ray14. The model achieved an AUC of 82.6% across 14 different thoracic diseases.

##### Mass

A lung mass is defined as a spot or abnormal area in the lungs larger than 3 cm (about 1.5 inches) in size.

On chest radiographs, Liang et al. [180] evaluated the diagnostic performance of a deep learning-based system for the detection of clinically significant lung nodules/masses. They used the ChestX-ray14 dataset for 100 patients with 47 mass images and 53 images without mass. They used four algorithms to detect pulmonary nodules/masses: heat map, abnormal probability, nodule probability, and mass probability. They used the QUIBIM Chest X-ray Classifier app module that assists radiologists in dealing with the vast amounts of chest radiographs. Chest radiographs are generated in health centers every day, by prioritizing potentially problematic instances. The Chest Radiograph Module is a collection of 14 pathology-specific 19-layer convolutional neural networks, followed by a fully connected layer that takes a chest radiograph and generates a likelihood of disease as well as heat maps indicating the areas of the image that are most symptomatic of chest disease. For pulmonary nodule/mass detection, the mass probability algorithm exhibited the best predictive performance with a sensitivity of 76.6%, AUC 91.6%, and specificity of 88.68%.

Li et al. [205] presented a faster Region-based convolutional neural network (RCNN) pre-trained on ResNet to diagnose lung mass disease. They used the JSRT dataset. The model achieved an accuracy of 53.38%.

## 4. Discussion

After analyzing these data from previous studies, we present the trend analysis of thoracic diseases detection recently through the following attributes, the analysis of the trend image type, transfer learning, data augmentation, deep learning model, and an ensemble classifier, respectively.

**Figure 9:**
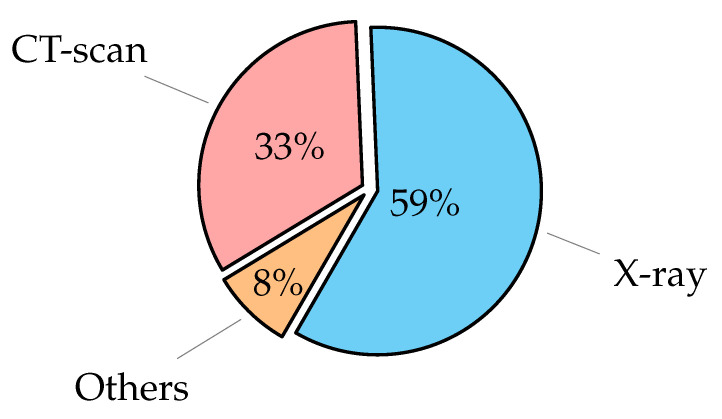
Image type of thoracic diseases distribution using deep learning in the recent years.

X-ray images were used in the majority of studies (59%) followed by CT scans (33%) as shown in Figure 9, and this is because it is cheaper, has a simple technique, has lower radiation compared to CT scans, and is widely used by radiologists to identify cracks, infection levels, and identify abnormal cases. However, it does not provide 3D information.

**Figure 10:**
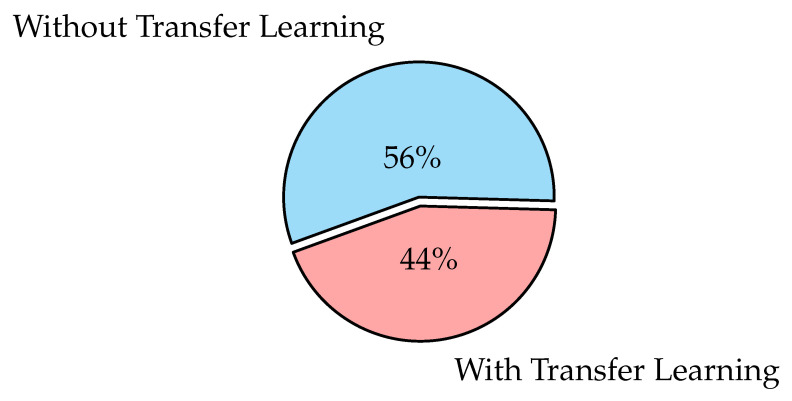
Transfer Learning distribution using deep learning aided thoracic diseases detection in the recent years.

In the majority of the research included in this review, DL models perform excellently when trained and tested on carefully selected datasets, including one or more classes of disease. The use of transfer learning has grown in popularity as shown in Figure 10. Transfer learning enables the use of features learned while training for a previous task to be applied to a new task, which improves classification accuracy. This could be because the model was trained on a larger number of images, making it more generalized.

**Figure 11:**
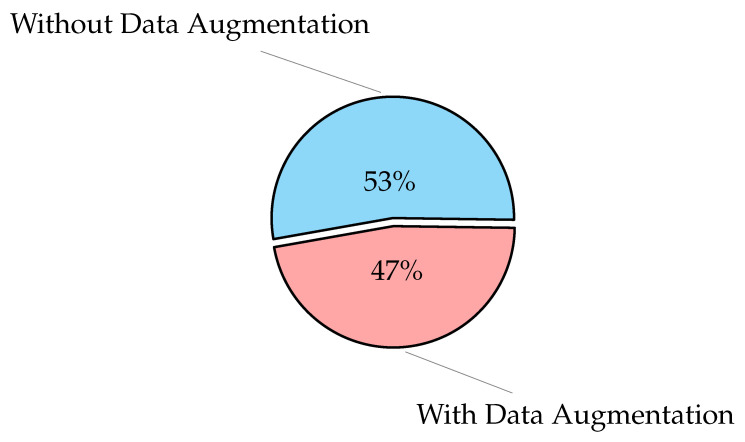
Data Augmentation distribution using deep learning aided thoracic diseases detection in the recent years.

When there is a limited number of data or the emergence of a new disease, such as the recent pandemic of COVID-19 disease. Thus, the data are in a higher class than another, which leads to the model becoming biased. Data Augmentation solved this problem. Data augmentation has the ability to improve the model’s performance and image quality when it is employed. As a result, the number of works that use data augmentation increased over time. Figure 11 illustrates the majority usage of data augmentation in this survey.

**Figure 12:**
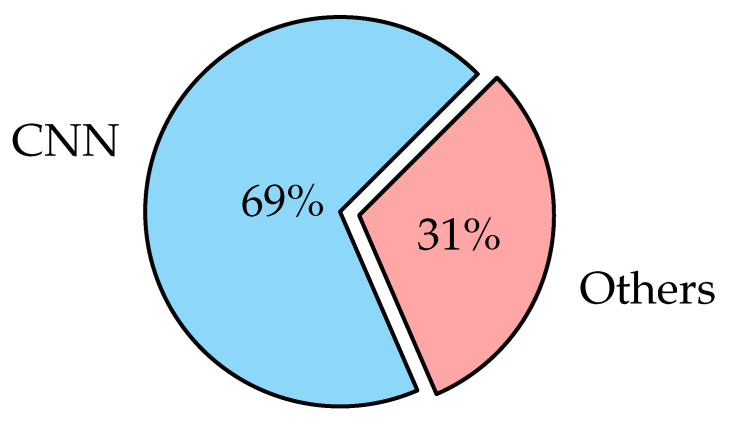
Model type distribution using deep learning aided thoracic diseases detection in the recent years.

In recent years, CNN has been the most used deep learning algorithm, as shown in Figure 12. More research may show that CNN is preferable to other deep learning algorithms for detecting thoracic diseases. This is due to CNN’s sturdiness, automatic feature extraction, and ability to achieve high classification accuracy.

**Figure 13:**
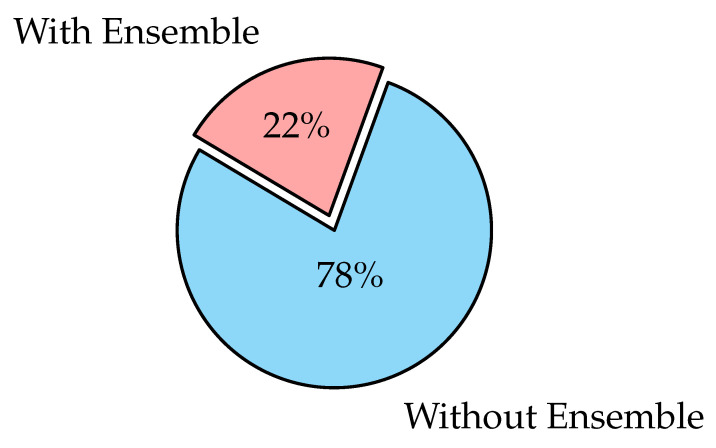
Ensemble distribution using deep learning aided thoracic diseases detection in the recent years.

Despite the fact that ensemble is a less popular technique, as shown in Figure 13, studies that used it reported superior detection performance than those that did not. This study shows that the use of an ensemble classifier to detect lung illness is still underused. The types of ensembles presented in the research are as follows: stacking, boosting, averaging, majority voting, and multi-scale ensemble module. Ensemble models can generate better predictions and accomplish better results than any single contributing model, and can reduce the spread or dispersion of the predictions.

This research presented several thoracic diseases that can automatically detect using deep learning, namely pneumonia, COVID-19, edema, lesion, cohesion, fibrosis, emphysema, atelectasis, asthma, asbestos signs, cardiomegaly, heart failure, chronic obstructive pulmonary disease, pleural thickening, fracture, lung metastasis, hernia, pneumothorax, mass, tuberculosis, and infiltration.

Segmentation of lung may increase the performance of the model. Therefore, some research uses it in some diseases as in [101].

## 5. Critical Analysis

There are four major difficulties/issues in the papers we presented: data imbalance, image size handling, dataset availability, and high correlation of errors when employing ensemble techniques:

(i) Data Imbalance: occurs when completing classification training. The resulting model will be biased if one class has a lot more samples than the other. It is preferable if each class has the same number of images. Therefore, researchers use the data augmentation technique to avoid this problem;

(ii) Image Size Handling: most studies reduced the original image size during training to save computing costs. Training a very complex model with the original image size is incredibly computationally expensive, and even with the most powerful GPU hardware, it takes a long time;

(iii) Dataset Availability: for training purposes, thousands of images of each class should be collected. This is carried out in order to create a more accurate classifier. The amount of available training data is generally less than optimal due to the limited number of datasets available. As a result, researchers are looking for new ways to produce a good classifier;

(iv) When employing ensemble approaches, there is a high correlation of errors: For an ensemble of classifiers to perform well, they must make a variety of errors. The correlation between the base classifiers employed should be very low. In other words, the base classifiers are supposed to work together to give better classification results. In the majority of the experiments surveyed, only classifiers with similar selected features were combined. As a result, the base classifier’s correlation errors are high.

Open issues that must be considered in order to improve the efficiency of deep learning models based thoracic diseases diagnosis:

- Publicize datasets, so researchers would have access to more data and the classifiers developed would be more accurate;
- Efforts can be focused on investigating several features. When employing ensemble approaches, this can help address the issue of high error correlation. As more features are added, the number of contrasts increases and the model’s accuracy improves. The results are often better when merging multiple versions;
- Using ensemble learning, especially in multi-classifications, to improve the accuracy of model detection and reduce training time;
- The majority of the models discussed in this analysis classify rather than localize or segment abnormalities, and this is an area that can be explored further;
- Unsupervised learning approaches like generative adversarial networks and variational autoencoders are being used by numerous researchers to investigate automated data curation.

## 6. Conclusions and Future Work

Medical practitioners and computer scientists all over the world are working collaboratively to develop effective techniques to diagnose thoracic diseases and track them by using AI-based methods. This paper provides a literature review of recent thoracic disease diagnosis and prediction research which involves the use of AI techniques. This research introduced a new classification of thoracic diseases from the medical point of view. It covered many different thoracic diseases, including COVID-19. A comprehensive survey of diseases belonging to this classification was made in terms of image type, the dataset used, model type, ensemble techniques, results, and open issues. Other researchers may use the classification provided to plan their contributions and research activities. A possible future approach could lead to increased efficiency and an increase in the number of applications for the detection of thoracic diseases with the help of AI.

The suggested future work is the use of multi-modality data, including medical visual data and patient health information, to verify the severity of the disease. Fusion methods will play a pivotal role in determining the severity score of the disease and overcoming the varied nature of the data utilized. The future of this work is to employ ML and/or DL algorithms to investigate various fusion techniques to achieve more accurate results and use recent models such as vision transformers (ViT), hybrid models, or explainable artificial intelligence (XAI) in the diagnosis of these diseases.

## Figures and Tables

**Table 1:** ML approaches summary.

Method	Advantage	Disadvantage
Supervised Learning	It performs classification and regression tasks. It exists notions of the output along the learning process.	It requires a labeled dataset.
Unsupervised Learning	It does not require a training data to be labeled. Classification task is fast.	There are no notions of the output along the learning process.
Semi-Supervised Learning	Builds a model through a mix of labeled and unlabeled data. Reduced training dataset.	Computationally complex.
Reinforcement Learning	Can gain experience and feedbacks (rewards) from their actions which help them to improve their results.	needs large datasets to make better benchmarks and decisions.

## Data Availability

Not applicable.

## References

[1] Kumar S., Singh P., Ranjan M. A review on deep learning based pneumonia detection systems. Proceedings of the 2021 International Conference on Artificial Intelligence and Smart Systems (ICAIS).

[2] Creek J. Lung Disease: Medlineplus Medical Encyclopedia. https://medlineplus.gov/ency/article/000066.htm.

[3] Omar S. Chest Diseases: Your Comprehensive Guide. www.webteb.com/articles/23328.

[4] EBC World Pneumonia Day. https://stoppneumonia.org/latest/world-pneumonia-day/.

[5] WHO Pneumonia. https://www.who.int/news-room/fact-sheets/detail/pneumonia.

[6] Team I. Coronavirus Cases. https://www.worldometers.info/coronavirus/.

[7] Rag C. Global Future of Imaging. https://www.bir.org.uk/get-involved/world-partner-network/global-future-of-imaging.aspx.

[8] Çallı E., Sogancioglu E., van Ginneken B., van Leeuwen K.G., Murphy K. (2021). Deep learning for chest X-ray analysis: A survey. Med. Image Anal..

[9] Haleem A., Javaid M., Khan I.H. (2019). Current status and applications of Artificial Intelligence (AI) in medical field: An overview. Curr. Med. Res. Pract..

[10] Davenport T., Kalakota R. The Potential for Artificial Intelligence in Healthcare. https://www.ncbi.nlm.nih.gov/pmc/articles/PMC6616181/.

[11] Pugliese R., Regondi S., Marini R. (2021). Machine learning-based approach: Global trends, research directions, and regulatory standpoints. Data Sci. Manag..

[12] Council of Europe Ai and Control of COVID-19 Coronavirus. https://www.coe.int/en/web/artificial-intelligence/ai-and-control-of-covid-19-coronavirus.

[13] Watson I., Jeong S., Hollingsworth J. How this South Korean Company Created Coronavirus Test Kits in Three Weeks. https://edition.cnn.com/2020/03/12/asia/coronavirus-south-korea-testing-intl-hnk/index.html.

[14] Baidu B. How Baidu is Bringing AI to the Fight against Coronavirus. https://www.technologyreview.com/2020/03/11/905366/how-baidu-is-bringing-ai-to-the-fight-against-coronavirus/.

[15] Mishra R.K., Reddy G.Y., Pathak H. (2021). The understanding of Deep Learning: A Comprehensive Review. Math. Probl. Eng..

[16] Lundervold A.S., Lundervold A. (2019). An overview of deep learning in medical imaging focusing on MRI. Z. Med. Phys..

[17] Krawczyk B., Minku L.L., Gama J., Stefanowski J., Woźniak M. (2017). Ensemble learning for data stream analysis: A survey. Inf. Fusion.

[18] Najafabadi M.M., Villanustre F., Khoshgoftaar T.M., Seliya N., Wald R., Muharemagic E. (2015). Deep learning applications and challenges in Big Data Analytics. J. Big Data.

[19] Das P.K., Meher S. (2021). An efficient deep Convolutional Neural Network based detection and classification of Acute Lymphoblastic Leukemia. Expert Syst. Appl..

[20] Das P., Meher S., Panda R., Abraham A. (2019). A Review of Automated Methods for the Detection of Sickle Cell Disease. IEEE Rev. Biomed. Eng..

[21] Das P., Pradhan A., Meher S. (2021). Detection of Acute Lymphoblastic Leukemia Using Machine Learning Techniques. Machine Learning, Deep Learning and Computational Intelligence for Wireless Communication.

[22] Das P., Meher S. Transfer Learning-Based Automatic Detection of Acute Lymphocytic Leukemia. Proceedings of the 2021 National Conference on Communications (NCC).

[23] Das P., Diya V., Meher S., Panda R., Abraham A. (2022). A Systematic Review on Recent Advancements in Deep and Machine Learning Based Detection and Classification of Acute Lymphoblastic Leukemia. IEEE Access.

[24] Das P.K., Nayak B., Meher S. (2022). A lightweight deep learning system for automatic detection of blood cancer. Measurement.

[25] Rajagopal R., Karthick R., Meenalochini P., Kalaichelvi T. (2023). Deep Convolutional Spiking Neural Network optimized with Arithmetic optimization algorithm for lung disease detection using chest X-ray images. Biomed. Signal Process. Control.

[26] Gao S., Lima D. (2022). A review of the application of deep learning in the detection of Alzheimer’s disease. Int. J. Cogn. Comput. Eng..

[27] EL-Geneedy M., Moustafa H.E.D., Khalifa F., Khater H., AbdElhalim E. (2023). An MRI-based deep learning approach for accurate detection of Alzheimer’s disease. Alex. Eng. J..

[28] Li Q., Zhang Y., Liang H., Gong H., Jiang L., Liu Q., Shen L. (2021). Deep learning based neuronal soma detection and counting for Alzheimer’s disease analysis. Comput. Methods Programs Biomed..

[29] Sultan S. Limitations of Artificial Intelligence. https://scholarworks.rit.edu/cgi/viewcontent.cgi?article=12113&context=theses.

[30] Kumar Y., Koul A., Singla R., Ijaz M.F. (2022). Artificial intelligence in disease diagnosis: A systematic literature review, synthesizing framework and future research agenda. J. Ambient. Intell. Humaniz. Comput..

[31] Victory L.R., Ervin K.M., Ridge C.A. (2020). Imaging in chest disease. Medicine.

[32] Ayan E., Ünver H.M. Diagnosis of Pneumonia from Chest X-ray Images Using Deep Learning. Proceedings of the 2019 Scientific Meeting on Electrical-Electronics Biomedical Engineering and Computer Science (EBBT).

[33] Ng K.H., Rehani M.M. (2006). X ray imaging goes digital. BMJ.

[34] Thompson W., Hudnut H., Russo P., Brown F., Mosley K. (1961). A review and study of cardiovascular disease screening with the miniature chest X-ray. J. Chronic Dis..

[35] Bharati S., Podder P., Mondal M.R.H. (2020). Hybrid deep learning for detecting lung diseases from X-ray images. Inform. Med. Unlocked.

[36] Saxena S., Jena B., Gupta N., Das S., Sarmah D., Bhattacharya P., Nath T., Paul S., Fouda M.M., Kalra M. (2022). Role of Artificial Intelligence in Radiogenomics for Cancers in the Era of Precision Medicine. Cancers.

[37] Jena B., Saxena S., Nayak G.K., Balestrieri A., Gupta N., Khanna N.N., Laird J.R., Kalra M.K., Fouda M.M., Saba L. (2022). Brain Tumor Characterization Using Radiogenomics in Artificial Intelligence Framework. Cancers.

[38] Soffer S., Morgenthau A.S., Shimon O., Barash Y., Konen E., Glicksberg B.S., Klang E. (2022). Artificial Intelligence for Interstitial Lung Disease Analysis on Chest Computed Tomography: A Systematic Review. Acad. Radiol..

[39] Dicente Cid Y., Liauchuk V., Klimuk D., Tarasau A., Kovalev V., Müller H. (2019). Overview of ImageCLEFtuberculosis 2019—Automatic CT-based Report Generation and Tuberculosis Severity Assessment. CLEF (Working Notes).

[40] Kassem M.N., Masallat D.T. (2021). Clinical application of chest computed tomography (CT) in detection and characterization of Coronavirus (COVID-19) pneumonia in adults. J. Digit. Imaging.

[41] Gurcan M., Boucheron L., Can A., Madabhushi A., Rajpoot N., Yener B. (2009). Histopathological Image Analysis: A Review. IEEE Rev. Biomed. Eng..

[42] Coudray N., Ocampo P., Sakellaropoulos T., Narula N., Snuderl M., Fenyö D., Moreira A., Razavian N., Tsirigos A. (2018). Classification and mutation prediction from non-small cell lung cancer histopathology images using deep learning. Nat. Med..

[43] He L., Long L.R., Antani S., Thoma G.R. (2012). Histology image analysis for carcinoma detection and grading. Comput. Methods Programs Biomed..

[44] Shah M., Mishra S., Yadav V., Chauhan A., Sarkar M., Sharma S., Rout C. (2017). Ziehl-Neelsen sputum smear microscopy image database: A resource to facilitate automated bacilli detection for tuberculosis diagnosis. J. Med. Imaging.

[45] Kant S., Srivastava M.M. Towards Automated Tuberculosis detection using Deep Learning. Proceedings of the 2018 IEEE Symposium Series on Computational Intelligence (SSCI).

[46] Das P., Ganguly S., Mandal B. (2019). Sputum Smear Microscopy in tuberculosis: It is still relevant in the era of molecular diagnosis when seen from the Public Health Perspective. Biomed. Biotechnol. Res. J. (BBRJ).

[47] ISTC International Standards for Tuberculosis Care. https://theunion.org/technical-publications/international-standards-for-tuberculosis-care%:%:text=The%International%Standards%of%Tuberculosis,%and%economic%losses%from%TB.

[48] Ishida M., Kato S., Sakuma H. (2009). Cardiac MRI in ischemic heart disease. Circ. J..

[49] Wang X., Peng Y., Lu L., Lu Z., Bagheri M., Summers R.M. ChestX-ray8: Hospital-Scale Chest X-ray Database and Benchmarks on Weakly-Supervised Classification and Localization of Common Thorax Diseases. Proceedings of the 2017 IEEE Conference on Computer Vision and Pattern Recognition (CVPR).

[50] Chowdhury M., Rahman T., Khandakar A., Mazhar R., Kadir M., Mahbub Z., Islam K., Khan M.S., Iqbal A., Al-Emadi N. (2020). Can AI help in screening Viral and COVID-19 pneumonia?. IEEE Access.

[51] Cohen J., Morrison P., Dao L. (2020). COVID-19 Image Data Collection. arXiv.

[52] Bustos A., Pertusa A., Salinas J.M., de la Iglesia-Vayá M. (2020). PadChest: A large chest X-ray image dataset with multi-label annotated reports. Med. Image Anal..

[53] Irvin J., Rajpurkar P., Ko M., Yu Y., Ciurea-Ilcus S., Chute C., Marklund H., Haghgoo B., Ball R., Shpanskaya K. Chexpert: A large chest radiograph dataset with uncertainty labels and expert comparison. Proceedings of the AAAI Conference on Artificial Intelligence.

[54] Wang L., Wong A. (2020). COVID-Net: A Tailored Deep Convolutional Neural Network Design for Detection of COVID-19 Cases from Chest Radiography Images. Sci. Rep..

[55] Rahman T., Khandakar A., Qiblawey Y., Tahir A., Kiranyaz S., Abul Kashem S.B., Islam M.T., Al Maadeed S., Zughaier S.M., Khan M.S. (2021). Exploring the effect of image enhancement techniques on COVID-19 detection using chest X-ray images. Comput. Biol. Med..

[56] Johnson A.E., Pollard T.J., Berkowitz S.J., Greenbaum N.R., Lungren M.P., Deng C.Y., Mark R.G., Horng S. (2019). Mimic-CXR, a de-identified publicly available database of chest radiographs with free-text reports. Sci. Data.

[57] Shiraishi J., Katsuragawa S., Ikezoe J., Matsumoto T., Kobayashi T., Komatsu K.I., Matsui M., Fujita H., Kodera Y., Doi K. (2000). Development of a digital image database for chest radiographs with and without a lung nodule: Receiver operating characteristic analysis of radiologists’ detection of pulmonary nodules. Am. J. Roentgenol..

[58] Kermany D.S., Zhang K., Goldbaum M.H. (2018). Labeled Optical Coherence Tomography (OCT) and Chest X-ray Images for Classification. Mendeley Data.

[59] Rsna P. RSNA Pneumonia Detection Challenge. https://www.kaggle.com/c/rsna-pneumonia-detection-challenge.

[60] Pan I., Agarwal S., Merck D. (2019). Generalizable Inter-Institutional Classification of Abnormal Chest Radiographs Using Efficient Convolutional Neural Networks. J. Digit. Imaging.

[61] Grossman R., Heath A., Ferretti V., Varmus H., Lowy D., Kibbe W., Staudt L. (2016). Toward a Shared Vision for Cancer Genomic Data. N. Engl. J. Med..

[62] Kozlovski S., Liauchuk V., Dicente Cid Y., Tarasau A., Kovalev V., Müller H. Overview of ImageCLEFtuberculosis 2020-Automatic CT-based Report Generation. Proceedings of the CLEF 2020.

[63] Jaeger S., Candemir S., Antani S., Wáng Y.X.J., Lu P.X., Thoma G. (2014). Two public chest X-ray datasets for computer-aided screening of pulmonary diseases. Quant. Imaging Med. Surg..

[64] Shakouri S., Bakhshali M.A., Layegh P., Kiani B., Masoumi F., Nakhaei S., Mostafavi S. (2021). COVID19-CT-dataset: An open-access chest CT image repository of 1000+ patients with confirmed COVID-19 diagnosis. BMC Res. Notes.

[65] Costa M.G.F., Filho C.F.F.C., Kimura A., Levy P.C., Xavier C.M., Fujimoto L.B. A sputum smear microscopy image database for automatic bacilli detection in conventional microscopy. Proceedings of the 2014 36th Annual International Conference of the IEEE Engineering in Medicine and Biology Society.

[66] Kermany D.S., Goldbaum M., Cai W., Valentim C.C., Liang H., Baxter S.L., McKeown A., Yang G., Wu X., Yan F. (2018). Identifying Medical Diagnoses and Treatable Diseases by Image-Based Deep Learning. Cell.

[67] Radau P., Lu Y., Connelly K., Paul G., Dick A., Wright G. (2009). Evaluation Framework for Algorithms Segmenting Short Axis Cardiac MRI. Card. MR Left Ventricle Segment. Chall..

[68] Edwards N., Oberti M., Thangudu R., Cai S., Mcgarvey P., Jacob S., Madhavan S., Ketchum K. (2015). The CPTAC data portal: A resource for cancer proteomics research. J. Proteome Res..

[69] Armato S., Mclennan G., Bidaut L., McNitt-Gray M., Meyer C., Reeves A., Zhao B., Aberle D., Henschke C., Hoffman E. (2011). The Lung Image Database Consortium (LIDC) and Image Database Resource Initiative (IDRI): A completed reference database of lung nodules on CT scans. Med. Phys..

[70] National Institutes of Health Chest X-ray Dataset, Kaggle NIH Chest X-rays. https://www.kaggle.com/nih-chest-xrays/data.

[71] ImageCLEF ImageCLEFmed Tuberculosis. https://www.imageclef.org/2019/medical/tuberculosis.

[72] ImageCLEF ImageCLEFmed Tuberculosis. https://www.imageclef.org/2020/medical/tuberculosis.

[73] JSRT Database JSRT Database: Japanese Society of Radiological Technology. http://db.jsrt.or.jp/eng.php.

[74] SK Tuberculosis A. Tuberculosis Chest X-ray Image Data Sets.—LHNCBC Abstract. https://lhncbc.nlm.nih.gov/publication/pub9931.

[75] Flavio T.I. TBImages—An Image Database of Conventional Sputum Smear Microscopy for Tuberculosis. http://www.tbimages.ufam.edu.br/.

[76] Larxel C. COVID-19 X rays. https://www.kaggle.com/andrewmvd/convid19-x-rays.

[77] Rahman T. COVID-19 Radiography Database. https://www.kaggle.com/tawsifurrahman/covid19-radiography-database.

[78] Kermany D. Large Dataset of Labeled Optical Coherence Tomography (OCT) and chest X-ray Images. https://data.mendeley.com/datasets/rscbjbr9sj/3.

[79] Raddar T. Tuberculosis Chest X-rays (Shenzhen). https://www.kaggle.com/raddar/tuberculosis-chest-xrays-shenzhen.

[80] Stanford ML Group Chexpert: A Large Dataset of Chest X-rays and Competition for Automated Chest X-ray Interpretation. https://stanfordmlgroup.github.io/competitions/chexpert/.

[81] BIMCV. https://bimcv.cipf.es/bimcv-projects/padchest/.

[82] Genomic Data Commons Data Portal. https://portal.gdc.cancer.gov/.

[83] Cohen J.P. IEEE8023/COVID-Chestxray-Dataset: We Are Building an Open Database of COVID-19 Cases with Chest X-ray or CT Images. https://github.com/ieee8023/covid-chestxray-dataset.

[84] Sajid N. COVID-19 Patients Lungs X ray Images 10000. https://www.kaggle.com/nabeelsajid917/covid-19-x-ray-10000-images.

[85] Mooney P. Chest X-ray Images (Pneumonia). https://www.kaggle.com/paultimothymooney/chest-xray-pneumonia.

[86] UCSD-AI4H COVID-CT/README.md. https://github.com/UCSD-AI4H/COVID-CT/blob/c224644822838e70b8f13b4ba90aa239ced992f7/README.md.

[87] Joinup C. Open Data. https://joinup.ec.europa.eu/collection/digital-response-covid-19/open-data.

[88] Vendt B. Data from LIDC-IDRI. https://wiki.cancerimagingarchive.net/display/Public/LIDC-IDRI.

[89] Zhao A. COVIDx CXR-2. https://www.kaggle.com/andyczhao/covidx-cxr2?select=competition_test.

[90] Berryman S. CPTAC-LUAD. https://wiki.cancerimagingarchive.net/pages/viewpage.action?pageId=33948253.

[91] Hussaini S. Sunnybrook Cardiac MRI. https://www.kaggle.com/salikhussaini49/sunnybrook-cardiac-mri.

[92] Johnson A., Pollard T., Mark R., Berkowitz S., Horng S. Mimic-CXR Database. https://physionet.org/content/mimic-cxr/2.0.0/.

[93] Domingos P. (2012). A Few Useful Things to Know about Machine Learning. Commun. ACM.

[94] El Asnaoui K., Chawki Y., Idri A. (2021). Automated methods for detection and classification pneumonia based on X-ray images using Deep Learning. Stud. Big Data.

[95] Mikołajczyk A., Grochowski M. Data augmentation for improving deep learning in image classification problem. Proceedings of the 2018 International Interdisciplinary PhD Workshop (IIPhDW).

[96] Zheng Z., Cai Y., Li Y. (2015). Oversampling method for imbalanced classification. Comput. Inform..

[97] Salimans T., Goodfellow I., Zaremba W., Cheung V., Radford A., Chen X. Improved Techniques for Training GANs. Proceedings of the 30th International Conference on Neural Information Processing Systems.

[98] Lin C.H., Lin C.J., Li Y.C., Wang S.H. (2021). Using Generative Adversarial Networks and Parameter Optimization of Convolutional Neural Networks for Lung Tumor Classification. Appl. Sci..

[99] He H., Bai Y., Garcia E.A., Li S. ADASYN: Adaptive synthetic sampling approach for imbalanced learning. Proceedings of the 2008 IEEE International Joint Conference on Neural Networks (IEEE World Congress on Computational Intelligence).

[100] Sogancioglu E., Murphy K., Calli E., Scholten E.T., Schalekamp S., Van Ginneken B. (2020). Cardiomegaly Detection on Chest Radiographs: Segmentation Versus Classification. IEEE Access.

[101] Rahman T., Khandakar A., Kadir M.A., Islam K.R., Islam K.F., Mazhar R., Hamid T., Islam M.T., Kashem S., Mahbub Z.B. (2020). Reliable tuberculosis detection using chest X-ray with deep learning, segmentation and visualization. IEEE Access.

[102] van der Velden B.H., Kuijf H.J., Gilhuijs K.G., Viergever M.A. (2022). Explainable artificial intelligence (XAI) in deep learning-based medical image analysis. Med. Image Anal..

[103] Yamashita R., Nishio M., Do R., Togashi K. (2018). Convolutional neural networks: An overview and application in radiology. Insights Into Imaging.

[104] O’Shea K., Nash R. (2015). An Introduction to Convolutional Neural Networks. arXiv.

[105] Ker J., Wang L., Rao J., Lim T. (2018). Deep Learning Applications in Medical Image Analysis. IEEE Access.

[106] Xu Z., Zhang H., Li N., Zhang L. (2017). Building extraction from high resolution SAR imagery based on deep neural networks. Remote Sens. Lett..

[107] Pandey R., Sahai A., Kashyap H., Pandey R., Khatri S.K., kumar Singh N., Verma P. (2022). Chapter 13—Implementing convolutional neural network model for prediction in medical imaging. Artificial Intelligence and Machine Learning for EDGE Computing.

[108] Alzubaidi L., Zhang J., Humaidi A.J., Al-Dujaili A., Duan Y., Al-Shamma O., Santamaría J., Fadhel M.A., Al-Amidie M., Farhan L. (2021). Review of Deep Learning: Concepts, CNN Architectures, challenges, applications, Future Directions. J. Big Data.

[109] Shokraei Fard A., Reutens D.C., Vegh V. (2022). From CNNs to GANs for cross-modality medical image estimation. Comput. Biol. Med..

[110] DiPietro R., Hager G.D., Zhou S.K., Rueckert D., Fichtinger G. (2020). Chapter 21-Deep learning: RNNs and LSTM. Handbook of Medical Image Computing and Computer Assisted Intervention.

[111] Sharkawy A.N. (2020). Principle of Neural Network and Its Main Types: Review. J. Adv. Appl. Comput. Math..

[112] Mithra K.S., Emmanuel W.R.S. (2019). Automated identification of mycobacterium bacillus from sputum images for tuberculosis diagnosis. Signal Image Video Process..

[113] Hinton G., Osindero S., Teh Y.W. (2006). A Fast Learning Algorithm for Deep Belief Nets. Neural Comput..

[114] Voulodimos A., Doulamis N., Doulamis A., Protopapadakis E. (2018). Deep Learning for Computer Vision: A brief review. Comput. Intell. Neurosci..

[115] Koo K., Min G., Kim J., Park J., Kim J., Ahn H., Min M., Kim J., Chung B. (2019). 166—A multilayer perceptron artificial neural network model for predicting survival of patients with prostate cancer according to initial treatment strategy: Development of a web-based clinical decision support system. Eur. Urol. Suppl..

[116] Akkaya B., Çolakoğlu N. Comparison of Multi-class Classification Algorithms on Early Diagnosis of Heart Diseases. Proceedings of the y-BIS Conference 2019: Recent Advances in Data Science and Business Analytics.

[117] Lin W., Hasenstab K., Cunha G., Schwartzman A. (2020). Comparison of handcrafted features and convolutional neural networks for liver MR image adequacy assessment. Sci. Rep..

[118] Nanni L., Ghidoni S., Brahnam S. (2017). Handcrafted vs. non-handcrafted features for computer vision classification. Pattern Recognit..

[119] Wang C., Chen D., Hao L., Liu X., Zeng Y., Chen J., Zhang G. (2019). Pulmonary Image Classification Based on Inception-v3 Transfer Learning Model. IEEE Access.

[120] Baltruschat I., Nickisch H., Grass M., Knopp T., Saalbach A. (2019). Comparison of Deep Learning Approaches for Multi-Label Chest X-ray Classification. Sci. Rep..

[121] Ashhar S., Mokri S., Abd. Rahni A.A., Huddin A., Zulkarnain N., Azmi N., Mahaletchumy T. (2021). Comparison of deep learning convolutional neural network (CNN) architectures for CT lung cancer classification. Int. J. Adv. Technol. Eng. Explor..

[122] Mamalakis M., Swift A.J., Vorselaars B., Ray S., Weeks S., Ding W., Clayton R.H., Mackenzie L.S., Banerjee A. (2021). DenResCov-19: A Deep Transfer Learning Network for robust automatic classification of COVID-19, pneumonia, and tuberculosis from X-rays. Comput. Med. Imaging Graph..

[123] Waheed A., Goyal M., Gupta D., Khanna A., Al-Turjman F., Pinheiro P. (2020). CovidGAN: Data Augmentation using Auxiliary Classifier GAN for Improved Covid-19 Detection. IEEE Access.

[124] Mahmud T., Rahman M.A., Fattah S.A. (2020). CovXNet: A multi-dilation convolutional neural network for automatic COVID-19 and other pneumonia detection from chest X-ray images with transferable multi-receptive feature optimization. Comput. Biol. Med..

[125] Wang G., Li W., Zuluaga M.A., Pratt R., Patel P.A., Aertsen M., Doel T., David A.L., Deprest J., Ourselin S. (2018). Interactive Medical Image Segmentation Using Deep Learning With Image-Specific Fine Tuning. IEEE Trans. Med. Imaging.

[126] Dietterich T.G. (2000). Ensemble Methods in Machine Learning. Multiple Classifier Systems.

[127] Sirazitdinov I., Kholiavchenko M., Mustafaev T., Yuan Y., Kuleev R., Ibragimov B. (2019). Deep neural network ensemble for pneumonia localization from a large-scale chest X-ray database. Comput. Electr. Eng..

[128] Ammar A., Bouattane O., Youssfi M. (2021). Automatic cardiac cine MRI segmentation and heart disease classification. Comput. Med. Imaging Graph..

[129] Subasi A., Kadasa B., Kremic E. (2020). Classification of the Cardiotocogram Data for Anticipation of Fetal Risks using Bagging Ensemble Classifier. Procedia Comput. Sci..

[130] Vo D.M., Nguyen N.Q., Lee S.W. (2019). Classification of breast cancer histology images using incremental boosting convolution networks. Inf. Sci..

[131] Sun L., Mo Z., Yan F., Xia L., Shan F., Ding Z., Song B., Gao W., Shao W., Shi F. (2020). Adaptive feature selection guided Deep Forest for COVID-19 classification with chest CT. IEEE J. Biomed. Health Inform..

[132] Rajaraman S., Candemir S., Xue Z., Alderson P., Kohli M., Abuya J., Thoma G., Antani S. A novel stacked generalization of models for improved TB detection in chest radiographs. Proceedings of the 2018 40th Annual International Conference of the IEEE Engineering in Medicine and Biology Society (EMBC).

[133] Deb S.D., Jha R.K., Jha K., Tripathi P.S. (2022). A multi model ensemble based deep convolution neural network structure for detection of COVID19. Biomed. Signal Process. Control.

[134] Simonyan K., Zisserman A. (2015). Very Deep Convolutional Networks for Large-Scale Image Recognition. arXiv.

[135] Zhang Q., Wang H., Yoon S.W., Won D., Srihari K. (2019). Lung Nodule Diagnosis on 3D Computed Tomography Images Using Deep Convolutional Neural Networks. Procedia Manuf..

[136] Choudhary P., Hazra A. (2019). Chest disease radiography in twofold: Using convolutional neural networks and transfer learning. Evol. Syst..

[137] Abdar A.K., Sadjadi S.M., Soltanian-Zadeh H., Bashirgonbadi A., Naghibi M. Automatic detection of coronavirus (COVID-19) from chest CT images using VGG16-based deep-learning. Proceedings of the 2020 27th National and 5th International Iranian Conference on Biomedical Engineering (ICBME).

[138] Khatibi T., Shahsavari A., Farahani A. (2021). Proposing a novel multi-instance learning model for tuberculosis recognition from chest X-ray images based on CNNs, complex networks and stacked ensemble. Phys. Eng. Sci. Med..

[139] Dey N., Zhang Y.D., Rajinikanth V., Pugalenthi R., Raja N.S. (2021). Customized VGG19 architecture for pneumonia detection in chest X-rays. Pattern Recognit. Lett..

[140] Szegedy C., Liu W., Jia Y., Sermanet P., Reed S., Anguelov D., Erhan D., Vanhoucke V., Rabinovich A. Going deeper with convolutions. Proceedings of the 2015 IEEE Conference on Computer Vision and Pattern Recognition (CVPR).

[141] Nguyen L., Lin D., Lin Z., Cao J. Deep CNNs for microscopic image classification by exploiting transfer learning and feature concatenation. Proceedings of the 2018 IEEE International Symposium on Circuits and Systems (ISCAS).

[142] Szegedy C., Vanhoucke V., Ioffe S., Shlens J., Wojna Z. Rethinking the Inception Architecture for Computer Vision. Proceedings of the IEEE Conference on Computer Vision and Pattern Recognition.

[143] Ali L., Alnajjar F., Jassmi H., Gochoo M., Khan W., Serhani M. (2021). Performance Evaluation of Deep CNN-Based Crack Detection and Localization Techniques for Concrete Structures. Sensors.

[144] He K., Zhang X., Ren S., Sun J. Deep Residual Learning for Image Recognition. Proceedings of the 2016 IEEE Conference on Computer Vision and Pattern Recognition (CVPR).

[145] Ouyang X., Karanam S., Wu Z., Chen T., Huo J., Zhou X.S., Wang Q., Cheng J.Z. (2021). Learning Hierarchical Attention for Weakly-Supervised Chest X-ray Abnormality Localization and Diagnosis. IEEE Trans. Med. Imaging.

[146] Szegedy C., Ioffe S., Vanhoucke V., Alemi A. (2017). Inception-v4, Inception-ResNet and the Impact of Residual Connections on Learning. AAAI Conf. Artif. Intell..

[147] Wang S., Zhang Y.D. (2020). DenseNet-201-Based Deep Neural Network with Composite Learning Factor and Precomputation for Multiple Sclerosis Classification. Acm Trans. Multimed. Comput. Commun. Appl..

[148] Huang G., Liu Z., Van Der Maaten L., Weinberger K.Q. Densely Connected Convolutional Networks. Proceedings of the 2017 IEEE Conference on Computer Vision and Pattern Recognition (CVPR).

[149] Chen J., Zhang D., Suzauddola M., Zeb A. (2021). Identifying crop diseases using attention embedded MobileNet-V2 model. Appl. Soft Comput..

[150] Sandler M., Howard A., Zhu M., Zhmoginov A., Chen L.C. MobileNetV2: Inverted Residuals and Linear Bottlenecks. Proceedings of the 2018 IEEE/CVF Conference on Computer Vision and Pattern Recognition.

[151] Abdelbaki S., Sakli N., Sakli H. (2021). Classification and Predictions of Lung Diseases from Chest X-rays Using MobileNet V2. Appl. Sci..

[152] Chollet F. Xception: Deep Learning with Depthwise Separable Convolutions. Proceedings of the 2017 IEEE Conference on Computer Vision and Pattern Recognition (CVPR).

[153] Zoph B., Vasudevan V., Shlens J., Le Q.V. Learning transferable architectures for Scalable Image Recognition. Proceedings of the 2018 IEEE/CVF Conference on Computer Vision and Pattern Recognition.

[154] Yang J., Ren P., Zhang D., Chen D., Wen F., Li H., Hua G. Neural Aggregation Network for Video Face Recognition. Proceedings of the 2017 IEEE Conference on Computer Vision and Pattern Recognition (CVPR).

[155] Ronneberger O. (2017). Invited talk: U-net convolutional networks for biomedical image segmentation. Inform. Aktuell.

[156] Lamba H. Understanding Semantic Segmentation with UNET. https://towardsdatascience.com/understanding-semantic-segmentation-with-unet-6be4f42d4b47.

[157] Cui H., Yuwen C., Jiang L., Xia Y., Zhang Y. (2021). Multiscale attention guided U-Net architecture for cardiac segmentation in short-axis MRI images. Comput. Methods Programs Biomed..

[158] Dabass M., Vashisth S., Vig R. (2021). Attention-Guided deep atrous-residual U-Net architecture for automated gland segmentation in colon histopathology images. Inform. Med. Unlocked.

[159] Wu M., Chai Z., Qian G., Lin H., Wang Q., Wang L., Chen H. (2021). Development and Evaluation of a Deep Learning Algorithm for Rib Segmentation and Fracture Detection from Multicenter Chest CT Images. Radiol. Artif. Intell..

[160] Singh P., Singh N., Singh K.K., Singh A., Singh K.K., Elhoseny M., Singh A., Elngar A.A. (2021). Chapter 5—Diagnosing of disease using machine learning. Machine Learning and the Internet of Medical Things in Healthcare.

[161] Sharma N., Saba L., Khanna N.N., Kalra M.K., Fouda M.M., Suri J.S. (2022). Segmentation-Based Classification Deep Learning Model Embedded with Explainable AI for COVID-19 Detection in Chest X-ray Scans. Diagnostics.

[162] Suri J.S., Agarwal S., Chabert G.L., Carriero A., Paschè A., Danna P.S. (2022). COVLIAS 2.0-cXAI: Cloud-Based Explainable Deep Learning System for COVID-19 Lesion Localization in Computed Tomography Scans. Diagnostics.

[163] Suri J., Agarwal S., Chabert G., Carriero A., Paschè A., Danna P., Saba L., Mehmedović A., Faa G., Singh I. (2022). COVLIAS 1.0Lesion vs. MedSeg: An Artificial Intelligence Framework for Automated Lesion Segmentation in COVID-19 Lung Computed Tomography Scans. Diagnostics.

[164] Sakib S., Tazrin T., Fouda M.M., Fadlullah Z.M., Guizani M. (2020). DL-CRC: Deep Learning-Based Chest Radiograph Classification for COVID-19 Detection: A Novel Approach. IEEE Access.

[165] Sakib S., Fouda M.M., Md Fadlullah Z., Nasser N. On COVID-19 Prediction Using Asynchronous Federated Learning-Based Agile Radiograph Screening Booths. Proceedings of the ICC 2021—IEEE International Conference on Communications.

[166] Chauhan G., Liao R., Wells W., Andreas J., Wang X., Berkowitz S., Horng S., Szolovits P., Golland P. Joint modeling of chest radiographs and radiology reports for pulmonary edema assessment. Proceedings of the International Conference on Medical Image Computing and Computer Assisted Intervention—MICCAI.

[167] Kim D., Myong J.P., Han S.W. (2021). Classification of Asbestosis in CT Imaging Data Using Convolutional LSTM. Res. Sq..

[168] Behzadi-khormouji H., Rostami H., Salehi S., Derakhshande-Rishehri T., Masoumi M., Salemi S., Keshavarz A., Gholamrezanezhad A., Assadi M., Batouli A. (2020). Deep Learning, reusable and problem-based architectures for detection of consolidation on chest X-ray images. Comput. Methods Programs Biomed..

[169] Wang H., Xia Y. (2018). ChestNet: A Deep Neural Network for Classification of Thoracic Diseases on Chest Radiography. arXiv.

[170] Bao Y., Makady Y.H.A., Mahmoodi S. Automatic diagnosis of COPD in lung CT images based on multi-view DCNN. Proceedings of the 10th International Conference on Pattern Recognition, Applications and Methods, Institute of Communication and University of Lisbon.

[171] Guan Q., Huang Y., Zhong Z., Zheng Z., Zheng L., Yang Y. (2018). Diagnose like a Radiologist: Attention Guided Convolutional Neural Network for Thorax Disease Classification. arXiv.

[172] Christe A., Peters A., Drakopoulos D., Heverhagen J., Geiser T., Stathopoulou T., Christodoulidis S., Anthimopoulos M., Mougiakakou S., Ebner L. (2019). Computer-Aided Diagnosis of Pulmonary Fibrosis Using Deep Learning and CT Images. Investig. Radiol..

[173] Tomita K., Touge H., Sakai H., Sano H., Tohda Y. (2019). Deep learning facilitates the diagnosis of adult asthma. Allergol. Int..

[174] Gooßen A., Deshpande H., Harder T., Schwab E., Baltruschat I., Mabotuwana T., Cross N., Saalbach A. (2019). Deep Learning for Pneumothorax Detection and Localization in Chest Radiographs. arXiv.

[175] Peng L., Lin L., Hu H., Zhang Q., Li H., Chen Q., Wang D., Han X.H., Iwamoto Y., Chen Y.W. (2019). Multi-scale deep convolutional neural networks for emphysema classification and quantification. Intell. Syst. Ref. Libr..

[176] Duong L.T., Le N.H., Tran T.B., Ngo V.M., Nguyen P.T. (2021). Detection of tuberculosis from chest X-ray images: Boosting the performance with Vision Transformer and transfer learning. Expert Syst. Appl..

[177] Abiyev R.H., Ma’aitah M.K. (2018). Deep convolutional neural networks for chest diseases detection. J. Healthc. Eng..

[178] Nirschl J.J., Janowczyk A., Peyster E.G., Frank R., Margulies K.B., Feldman M.D., Madabhushi A. (2018). A deep-learning classifier identifies patients with clinical heart failure using whole-slide images of H&E Tissue. PLoS ONE.

[179] Mo S., Cai M. Deep Learning Based Multi-Label Chest X-ray Classification with Entropy Weighting Loss. Proceedings of the 2019 12th International Symposium on Computational Intelligence and Design (ISCID).

[180] Liang C.H., Liu Y.C., Wu M.T., Garcia-Castro F., Alberich-Bayarri A., Wu F.Z. (2020). Identifying pulmonary nodules or masses on chest radiography using deep learning: External validation and strategies to improve clinical practice. Clin. Radiol..

[181] Rachael Zimlich B. What You Need to Know about Lung Disease. https://www.verywellhealth.com/types-of-lung-disease-what-you-should-know-5207533.

[182] Smart J., Smart J. (1964). Chapter VIII—DIseases of the Lungs. A Synopsis of Respiratory Diseases.

[183] Liao R., Rubin J., Lam G., Berkowitz S.J., Dalal S., Wells W.M., Horng S., Golland P. (2019). Semi-Supervised Learning for Quantification of Pulmonary Edema in Chest X-ray Images. arXiv.

[184] Fu X., Liu T., Xiong Z., Smaill B.H., Stiles M.K., Zhao J. (2018). Segmentation of histological images and fibrosis identification with a convolutional neural network. Comput. Biol. Med..

[185] Bhatt A., Ganatra A., Kotecha K. (2021). COVID-19 pulmonary consolidations detection in chest X-ray using progressive resizing and transfer learning techniques. Heliyon.

[186] Chen S., Han Y., Lin J., Zhao X., Kong P. (2020). Pulmonary nodule detection on chest radiographs using balanced convolutional neural network and classic candidate detection. Artif. Intell. Med..

[187] Min Kim H., Ko T., Young Choi I., Myong J.P. (2022). Asbestosis diagnosis algorithm combining the lung segmentation method and deep learning model in computed tomography image. Int. J. Med. Inform..

[188] Ho T., Kim T., Kim W.J., Lee C.H., Chae K., Bak S., Kwon S., Jin G., Park E.K., Choi S. (2021). A 3D-CNN model with CT-based parametric response mapping for classifying COPD subjects. Sci. Rep..

[189] Chaudhary A., Hazra A., Prakash C. Diagnosis of Chest Diseases in X-ray images using Deep Convolutional Neural Network. Proceedings of the 2019 10th International Conference on Computing, Communication, and Networking Technologies (ICCCNT).

[190] Spyroglou I., Spöck G., Chatzimichail E., Rigas A., Paraskakis E. (2018). A Bayesian logistic regression approach in asthma persistence prediction. Epidemiol. Biostat. Public Health.

[191] Aboutalebi H., Pavlova M., Shafiee M.J., Sabri A., Alaref A., Wong A. (2021). COVID-Net CXR-S: Deep Convolutional Neural Network for Severity Assessment of COVID-19 Cases from Chest X-ray Images. Diagnostics.

[192] Allioui H., Mohammed M., Benameur N., Al-Khateeb B., Abdulkareem K., Zapirain B., Damaševičius R., Maskeliunas R. (2022). A Multi-Agent Deep Reinforcement Learning Approach for Enhancement of COVID-19 CT Image Segmentation. J. Pers. Med..

[193] El-Melegy M., Mohamed D., El Melegy T. Automatic detection of tuberculosis bacilli from microscopic sputum smear images using faster R-CNN, Transfer Learning and Augmentation. Proceedings of the Iberian Conference on Pattern Recognition and Image Analysis.

[194] Contributors H. What Is COPD?. https://health.howstuffworks.com/diseases-conditions/respiratory/what-is-copd.htm.

[195] Mohamed I., Fouda M.M., Hosny K.M. (2022). Machine Learning Algorithms for COPD Patients Readmission Prediction: A Data Analytics Approach. IEEE Access.

[196] WHO Chronic Obstructive Pulmonary Disease (COPD). https://www.who.int/news-room/fact-sheets/detail/chronic-obstructive-pulmonary-disease-(copd).

[197] Wallace G., Winter J., Winter J., Taylor A., Taylor T., Cameron R. (2009). Chest X-rays in COPD screening: Are they worthwhile?. Respir. Med..

[198] Park S., Lee S.M., Kim N., Choe J., Cho Y., Do K.H., Seo J.B. (2019). Application of deep learning-based computer-aided detection system: Detecting pneumothorax on chest radiograph after biopsy. Eur. Radiol..

[199] Pouraliakbar H., Maleki M., Alizadehasl A., Haghjoo M. (2022). Chapter 6—Chest Radiography in Cardiovascular Disease. Practical Cardiology.

[200] Teams W. Cardiovascular Diseases (CVDs). https://www.who.int/news-room/fact-sheets/detail/cardiovascular-diseases-(cvds).

[201] Candemir S., Rajaraman S., Thoma G., Antani S. Deep Learning for Grading Cardiomegaly Severity in Chest X-rays: An Investigation. Proceedings of the 2018 IEEE Life Sciences Conference (LSC).

[202] Wang Z., Chen X., Tan X., Yang L., Kannapur K., Vincent J.L., Kessler G.N., Ru B., Yang M. (2021). Using deep learning to identify high-risk patients with heart failure with reduced ejection fraction. J. Health Econ. Outcomes Res..

[203] Zhou Q.Q., Wang J., Tang W., Hu Z.C., Xia Z.Y., Li X.S., Zhang R., Yin X., Zhang H. (2020). Automatic Detection and Classification of Rib Fractures on Thoracic CT Using Convolutional Neural Network: Accuracy and Feasibility. Korean J. Radiol..

[204] Wang H., Wang S., Qin Z., Zhang Y., Li R., Xia Y. (2021). Triple attention learning for classification of 14 thoracic diseases using chest radiography. Med. Image Anal..

[205] Li Z., Li L. A novel method for lung masses detection and location based on deep learning. Proceedings of the 2017 IEEE International Conference on Bioinformatics and Biomedicine (BIBM).

